# Predicting the Risk of HIV Infection and Sexually Transmitted Diseases Among Men Who Have Sex With Men: Cross-Sectional Study Using Multiple Machine Learning Approaches

**DOI:** 10.2196/59101

**Published:** 2025-02-20

**Authors:** Bing Lin, Jiaxiu Liu, Kangjie Li, Xiaoni Zhong

**Affiliations:** 1 School of Public Health Chongqing Medical University Chongqing China; 2 Research Center for Medicine and Social Development Chongqing Medical University Chongqing China; 3 School of Medical Informatics Chongqing Medical University Chongqing China

**Keywords:** HIV, sexually transmitted diseases, men who have sex with men, machine learning, web application, risk stratification

## Abstract

**Background:**

Men who have sex with men (MSM) are at high risk for HIV infection and sexually transmitted diseases (STDs). However, there is a lack of accurate and convenient tools to assess this risk.

**Objective:**

This study aimed to develop machine learning models and tools to predict and assess the risk of HIV infection and STDs among MSM.

**Methods:**

We conducted a cross-sectional study that collected individual characteristics of 1999 MSM with negative or unknown HIV serostatus in Western China from 2013 to 2023. MSM self-reported their STD history and were tested for HIV. We compared the accuracy of 6 machine learning methods in predicting the risk of HIV infection and STDs using 7 parameters for a comprehensive assessment, ranking the methods according to their performance in each parameter. We selected data from the Sichuan MSM for external validation.

**Results:**

Of the 1999 MSM, 72 (3.6%) tested positive for HIV and 146 (7.3%) self-reported a history of previous STD infection. After taking the results of the intersection of the 3 feature screening methods, a total of 7 and 5 predictors were screened for predicting HIV infection and STDs, respectively, and multiple machine learning prediction models were constructed. Extreme gradient boost models performed optimally in predicting the risk of HIV infection and STDs, with area under the curve values of 0.777 (95% CI 0.639-0.915) and 0.637 (95% CI 0.541-0.732), respectively, demonstrating stable performance in both internal and external validation. The highest combined predictive performance scores of HIV and STD models were 33 and 39, respectively. Interpretability analysis showed that nonadherence to condom use, low HIV knowledge, multiple male partners, and internet dating were risk factors for HIV infection. Low degree of education, internet dating, and multiple male and female partners were risk factors for STDs. The risk stratification analysis showed that the optimal model effectively distinguished between high- and low-risk MSM. MSM were classified into HIV (predicted risk score <0.506 and ≥0.506) and STD (predicted risk score <0.479 and ≥0.479) risk groups. In total, 22.8% (114/500) were in the HIV high-risk group, and 43% (215/500) were in the STD high-risk group. HIV infection and STDs were significantly higher in the high-risk groups (*P*<.001 and *P*=.05, respectively), with higher predicted probabilities (*P*<.001 for both). The prediction results of the optimal model were displayed in web applications for probability estimation and interactive computation.

**Conclusions:**

Machine learning methods have demonstrated strengths in predicting the risk of HIV infection and STDs among MSM. Risk stratification models and web applications can facilitate clinicians in accurately assessing the risk of infection in individuals with high risk, especially MSM with concealed behaviors, and help them to self-monitor their risk for targeted, timely diagnosis and interventions to reduce new infections.

## Introduction

### HIV Infection and Sexually Transmitted Disease Situation of Chinese Men Who Have Sex With Men

Men who have sex with men (MSM) are at high risk of contracting HIV infection and other sexually transmitted diseases (STDs), and the number of infections is growing fast. The latest report released by the Joint United Nations Program for HIV and AIDS 2023 stated that HIV prevalence was 11 times higher among MSM compared to adults in the general population [1]. According to the results of the current large-scale systematic analysis study in China, the national HIV prevalence rate in the MSM population from 2001 to 2018 was 5.7%, compared to 0.037% in the general population in 2014 [2,3]. At the same time, the burden of other STDs (eg, syphilis, gonorrhea, and condyloma acuminata) among MSM was also high and increasing [4]. The prevalence of syphilis in the MSM population was as high as 35%, comprising both known and newly diagnosed cases [5]. The prevalence of gonorrhea and chlamydia among MSM in Southern China has been reported to be as high as 12.5% and 18.2%, respectively [6,7]. In addition, these STDs have the potential to facilitate HIV transmission and infection [8]. Therefore, given the severe HIV infection and STD situation of Chinese MSM, improving risk perception strategies and developing accurate risk prediction tools can help to prevent and control these infections, thereby improving their quality of life.

### Using Machine Learning for Accurate Risk Assessment

Establishing accurate risk assessment and prediction tools for the MSM population is beneficial for clinical services to identify high-risk individuals and prioritize resources. Currently, existing models, such as multivariate logistic regression, Poisson regression, and Cox proportional hazards regression, have been widely used in the context of HIV infection or other STDs [9-11]. However, data structures that were often nonlinear, anomalous, and heterogeneous could lead to poor prediction performance. Despite the existence of traditional statistical methods, research exploring more sophisticated analytical approaches to enhance disease classification and predictive capabilities among the MSM population has been limited. Machine learning provides an innovative perspective on evaluating the risk of infections in population with high risk, allowing more flexibility in handling complex, nonlinear relationships between the variables, thus minimizing error [12]. Furthermore, machine learning is composed of a set of powerful algorithms that have the ability to acquire knowledge, predict outcomes, and review data. The use of these algorithms has attracted great attention in the development of patient-centered predictive models, which can provide valuable clinical insights [13]. However, currently, there are few studies using machine learning methods to predict the risk of HIV infection and STDs in China, and our study extended the literature in this context.

### Risk Prediction for Serving Clinical Applications

The ultimate goal of risk prediction is intended to serve clinical applications prioritizing the screening of high-risk individuals in need of intervention. However, if too many predictors were included in the prediction model, it would not be conducive to collecting and applying them in clinical situations. In addition, there were various feature selection methods, each offering unique advantages but also having inevitable drawbacks. If multiple methods were used, the likelihood of screening for excellent predictors could be increased. Therefore, when faced with a large number of variables or when trying to construct a predictive model with a small number of predictors, a *combined* feature screening method was necessary. This method could help to decrease the data dimensions, reduce the amount of computation, and increase the applicability while eliminating some irrelevant or redundant variables and improving the prediction effect [14]. In addition, due to the “black box” of machine learning algorithms, understanding why a specific prediction was made for an individual or how a particular characteristic of an individual influenced a prediction was challenging [15]. The lack of interpretability limited the widespread use of more robust machine learning methods to support medical decision-making [16]. The limited intuitive understanding of machine learning models was still a significant barrier to their implementation in health care due to their inability to reflect the true correlation between individual characteristics and predicted outcomes [17]. As a result, the ability to correctly interpret predictive model outputs is important for generating appropriate user trust, providing insights to improve the model, and supporting an understanding of the modeling process to leverage the potential and productivity benefits of machine learning approaches. Moreover, some studies have failed to effectively address the data imbalance issues in their datasets, and further efforts to address this imbalance may improve model performance [18].

### Developing Risk Screening Tools

To address the limitations of previous research, our study comprehensively considered feature selection, model interpretability, and strategies for handling class imbalance in the data. First, we applied a “combined” feature selection process using 3 methods, including univariate analysis, least absolute shrinkage and selection operator (LASSO), and Boruta, to select features and incorporate variables common to each method. At the same time, a series of data processing techniques and machine learning methods were used to identify individuals at risk for HIV infection and STDs in the MSM population. The risk factors for infection were analyzed, and interpretations were supported by the use of partial dependence plots (PDPs), which were designed to provide theoretical support for targeted screening strategies and risk management. Our study had a more comprehensive modeling strategy than previous studies. Moreover, our study did not incorporate difficult-to-obtain clinical information, such as blood-related, immunologic, or cytologic data. All predictors were relatively easy to assess, making them more suitable for widespread use in screening studies among the MSM population. Finally, regarding clinical applications, the deployment of machine learning predictions into web applications showed good potential and application value. The user-friendly web applications served as pioneering digital tools that have been widely used in managing diabetic blood glucose, analyzing gliomas, and predicting early mortality in patients with bone metastases; however, in MSM populations, the risk of HIV infection and STDs has had limited application [19-21]. At the same time, the MSM population may conceal their sexual behavior due to external pressures from family members, traditional social attitudes, and fear of discrimination and stigmatization [22]. Web applications can help MSM conduct real-time, interactive self-monitoring anytime, anywhere, serving the sexual minority individuals and helping to protect their privacy. These web applications are a simple, practical, and effective risk management tool that can be widely used by clinicians and MSM individuals, and web applications offer advantages such as time efficiency, user-friendliness, and privacy protection. In summary, our study aimed to use machine learning methods to develop accessible, user-friendly web applications and tools to provide valuable insights into the risk factors influencing HIV infection and STDs, as well as to tailor screening models for the MSM population to accurately predict the risk of HIV infection and STDs in this population.

## Methods

### Study Design and Participants

Our study was a multistage, multicenter cross-sectional survey conducted from 2013 to 2023, based on the key projects of infectious disease research funded by the Chinese Ministry of Science and Technology during the 12th Five-Year Plan and 13th Five-Year Plan periods. Because of the particularity of the MSM population as sexual minority individuals, we used a nonprobability sampling method to recruit MSM in Western China (ie, Chongqing, Xinjiang, Guangxi, and Sichuan) through collaboration with nongovernmental organizations.

Specifically, we advertised on websites, QQ (Shenzen Tencent Computer System), and WeChat (Tencent Holdings Ltd) groups, and we collaborated with local nongovernment organizations to provide information on HIV prevention, counseling, and testing for lesbians, gay men, bisexual, and transgender individuals on each platform. We used this approach to present the details of the study (ie, objectives, procedures, potential benefits, and risks) to the leaders to gain their support and, with their help, recruit participants from the organizations. MSM were included in our study to complete the questionnaire if they were aged ≥18 years, had sexual contact with a man in the past 12 months, and had negative or unknown HIV serostatus. Participants who met the requirements went to local hospitals or community centers with which we had collaborated, where our researchers conducted face-to-face questionnaires and performed the appropriate tests. If participants encountered any unclear terms, trained investigators explained their meaning to them. All questionnaires were subsequently checked for completeness and consistency.

### Outcomes

We tested MSM for HIV to determine whether they had experienced HIV infection. Those who were previously infected with STD among MSM were measured by the question, “In the last six months, have you been diagnosed by a doctor with the STD (for example, syphilis, gonorrhea, condyloma acuminatum, genital herpes, non-specific urethritis, and so on)?” On the basis of HIV test results, patients who obtained positive results were labeled as positive outcome indicators, and those who obtained nonpositive results were labeled as negative outcome indicators. Those who were previously infected with an STD were labeled as positive outcome indicators. and those who were never infected with an STD were labeled as negative outcome indicators. Predictive models were developed based on each of the 2 outcome indicators, HIV and STD.

### Predictors

A total of 24 variables were selected for the personal characteristics of MSM, including four main aspects: (1) demographic characteristics; (2) HIV-related knowledge, behavior, and risk perception; (3) sexual behavior–related characteristics; and (4) substance use. Among them, demographic characteristics included age, household registration location, ethnicity, degree of education, employment situation, marital status, and monthly disposable income. HIV-related knowledge, behaviors, and risk perception included HIV knowledge scores (≥11 was defined as “high level” [22]), HIV testing and counseling, and HIV risk perceptions. Sexual behavior–related characteristics included sexual role (ie, the insertive partner and the receptive partner), number of sexual partners, high-risk sex, and condom use. Substance use included internet dating (ie, finding sexual partners through the internet), alcohol use, commercial sexual services (eg, offering and receiving sexual services), and recreational drug use. We excluded samples with too much missing data or where the presence of HIV infection or STD could not be determined. After a preliminary analysis, all variables had a missing rate of <10%, so we used the multiple imputation method to fill in the missing values.

### Feature Selection

Redundant information in survey data from the MSM population may lead to suboptimal categorization of HIV infection or STD, as excessive dimensions in the data can reduce the accuracy and validity of the models [15]. Therefore, it is promising to use the “combined” feature selection method to select variables for the raw data. Univariate analysis, LASSO, and Boruta were used in our study. In this study, variables with *P*<.10 were included in the univariate analysis; variables with regression coefficients not equal to 0 were included in LASSO; and variables that were classified as confirmed or tentative were included, while rejected variables were excluded in Boruta. Using 3 different methods for feature selection, we ultimately included factors common to all these methods as predictors in the predictive models.

### Prediction Models

Our dataset showed a significant imbalance in the number of individuals with and without HIV and STDs. Data-level resampling techniques have been increasingly used to address unbalanced datasets due to its simplicity and ease of implementation. Therefore, our study used the synthetic minority oversampling technique to deal with the unbalanced dataset. This method oversampled the minority class samples in the boundary region, and it expanded the decision space of the minority class samples with little effect on the decision space of the majority class samples.

Logistic regression, decision tree, random forest, K-nearest neighbor, light gradient boosting machine, and extreme gradient boosting (XGBoost) were used to predict the risk of HIV infection or STDs in the MSM population. To train, construct, and evaluate the 6 predictive models, we divided 75% (1499/1999) of the data into a training set, 25% (500/1999) into a testing set, and used the data from the Sichuan MSM as an external validation set. Using the training data, a cross-validation grid search was conducted to determine the optimal hyperparameters for the predictive model. In this study, we used 7 standard performance metrics, including accuracy, balanced accuracy, sensitivity, specificity, positive predictive value, negative predictive value, and area under the receiver operating characteristic curve, to evaluate the classification performance of the prediction model. Meanwhile, 10-fold cross-validation was used to assess the stability of several machine learning prediction models. These evaluation metrics were calculated from a binary confusion matrix, and based on the results of previous studies, these metrics were ranked according to their predictive performance [21,23]. On the basis of each evaluation metric, the highest score was assigned to the best-performing model, followed by the next best-performing model. The total score of the model was the sum of the scores for each metric. The data were heat mapped using the *pheatmap* package in R (R Foundation for Statistical Computing).

### Model Interpretation

Interpretable analyses of the model included both variable importance ranking and PDP to identify key predictors of HIV infection or STDs in the MSM population. Feature importance refers to quantifying the contribution of each variable in the predictive model to the prediction outcome and ranking them accordingly, which reflects the magnitude of each predictor’s influence on the model. Our study used PDP to conduct an explanatory analysis of the predictive model, assessing the degree and direction of influence of each variable on the prediction outcome.

### Risk Stratification

The probability of the optimal model output was defined as the predicted risk score (PRS) [24]. The cutoff point could be used as a basis for risk definition, and based on this threshold, MSM were categorized into low-risk and high-risk individuals. MSM with a predicted probability less than the threshold were assigned to the low-risk group, while MSM with a predicted probability greater than or equal to the threshold were assigned to the high-risk group. This approach allowed differentiation of MSM based on predicted risk, facilitating targeted interventions and individualized management strategies.

### Web Applications

Using the algorithm of the optimal model in this study, we developed web-based calculators to predict the risk of HIV infection or STDs in the MSM population. The applications were composed of 3 main parts, which were the input box, the prediction result, and the result box. The input box included the predictors of the prediction model, and the user could input the values of the predictors independently according to the individual situation. This interactive panel allowed the user to customize the prediction based on specific individual characteristics. Clicking on the Predict button below the input box displayed the predicted outcome under different conditions, which was the probability of an individual MSM being infected with HIV or STD. This information could provide valuable insights for clinical decision-making, and web applications can predict outcomes under different conditions and save these multiple predictions in the results box.

### Sensitivity Analysis

According to the sexual behavior–related characteristics, MSM could be categorized into MSM and women (MSMW), MSM only (MSMO) [25], men who have sex with multiple men (MSMM), and non-MSMM [26]. Previous studies have found differences between MSMW and MSMO in HIV-related high-risk sexual behavior and HIV infection or STD prevalence [27]. At the same time, MSMM had a higher risk of infection than other men [28]. For these subgroups with different risks of infection, we included MSMW and MSMO as well as MSMM and non-MSMM into the optimal model to predict the risk of HIV infection or STDs in these subgroups. This was done to perform sensitivity analyses and validate the stability of the prediction model.

### Statistical Analysis

**Figure 1:**
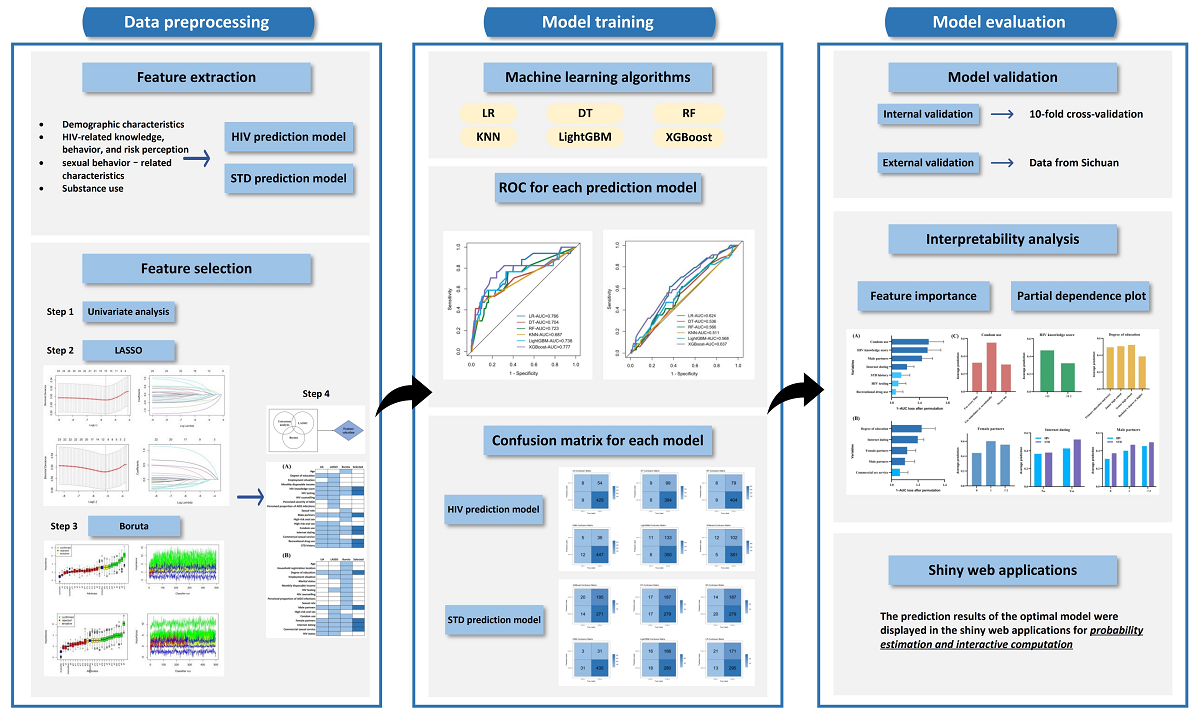
Flowchart of the data analysis and machine learning modeling process. DT: decision tree; KNN: K-nearest neighbor; LASSO: least absolute shrinkage and selection operator; LightGBM: light gradient boosting machine; LR: logistic regression; RF: random forest; ROC: receiver operating characteristic curve; STD: sexually transmitted disease; XGBoost: extreme gradient boosting.

For all statistical analyses, we used SAS (SAS Institute) and R software. Multiple imputation and univariate analysis were accomplished using SAS. For continuous variables that did not follow a normal distribution, we used nonparametric tests for comparison; for comparisons involving categorical variables, we used the chi-square test. If the expected count in 25% of the cells was <5, the chi-square test was deemed ineffective, and the Fisher exact probability test was used. LASSO, Boruta, machine learning algorithms, and plotting were performed using the *glmnet*, *Boruta*, *tidymodels*, and *ggplot* packages in R, respectively. The model interpretability analysis was performed using the *dalex* package for variable importance ranking and PDPs, and the web applications used the *shiny* package in R. The flowchart of the data analysis and machine learning modeling process is presented in Figure 1.

### Ethical Considerations

The study was approved by the Chongqing Medical University Ethics Committee (reference numbers 2012010 for 12th Five-Year Plan and 2019001 for 13th Five-Year Plan). Before participating in this research, the participants were fully informed about the purpose, significance, voluntary participation, and confidentiality of the research. Each participant signed an informed consent form and was also compensated with RMB 80 (US $10.90) for transportation and breakfast. The study data were anonymized and deidentified to ensure participant privacy and confidentiality. All images in the manuscript and Multimedia Appendix 1 were reviewed to ensure that individual participants and users cannot be identified.

## Results

### Overview

In total, we collected individual characteristics from 2345 MSM and excluded those who did not meet the requirements, resulting in 1999 (85.24%) MSM being included in the analyses (refer to Figure S1 in Multimedia Appendix 1 for a flowchart of the participant screening process). The percentage of HIV-positive MSM was 3.6% (72/1999), and the percentage of those who self-reported as previously infected with STD was 7.3% (146/1999). The basic demographic information of the 1999 MSM is shown in Table 1.

**Table 1:** Description of basic demographic variables for the surveyed population of men who have sex with men (N=1999).

Variables		Values, n (%)
**Age (y)**		
	18-25	147 (7.35)
	25-35	732 (36.62)
	35-45	689 (34.47)
	≥45	431 (21.56)
**Household registration location**		
	Urban	1430 (71.54)
	Rural	569 (28.46)
**Ethnicity**		
	Ethnic minority individuals	168 (8.4)
	Han	1831 (91.6)
**Degree of education**		
	Primary education and lower	42 (2.1)
	Junior high school	170 (8.5)
	Senior high school	955 (47.77)
	Bachelor’s degree or higher	792 (39.62)
**Employment**		
	Unemployed	177 (8.85)
	Employed	1591 (79.59)
	Student	231 (11.55)
**Marital status**		
	Unmarried	1685 (84.29)
	Married	314 (15.71)
**Monthly disposable income (RMB)**		
	1000 (US $137.20) to 3000 (US $411.50)	811 (40.57)
	3000 (US $411.50) to 10,000 (US $1371.60)	1088 (54.43)
	≥10,000 (US $1371.60)	100 (5)

### Feature Selection

**Figure 2:**
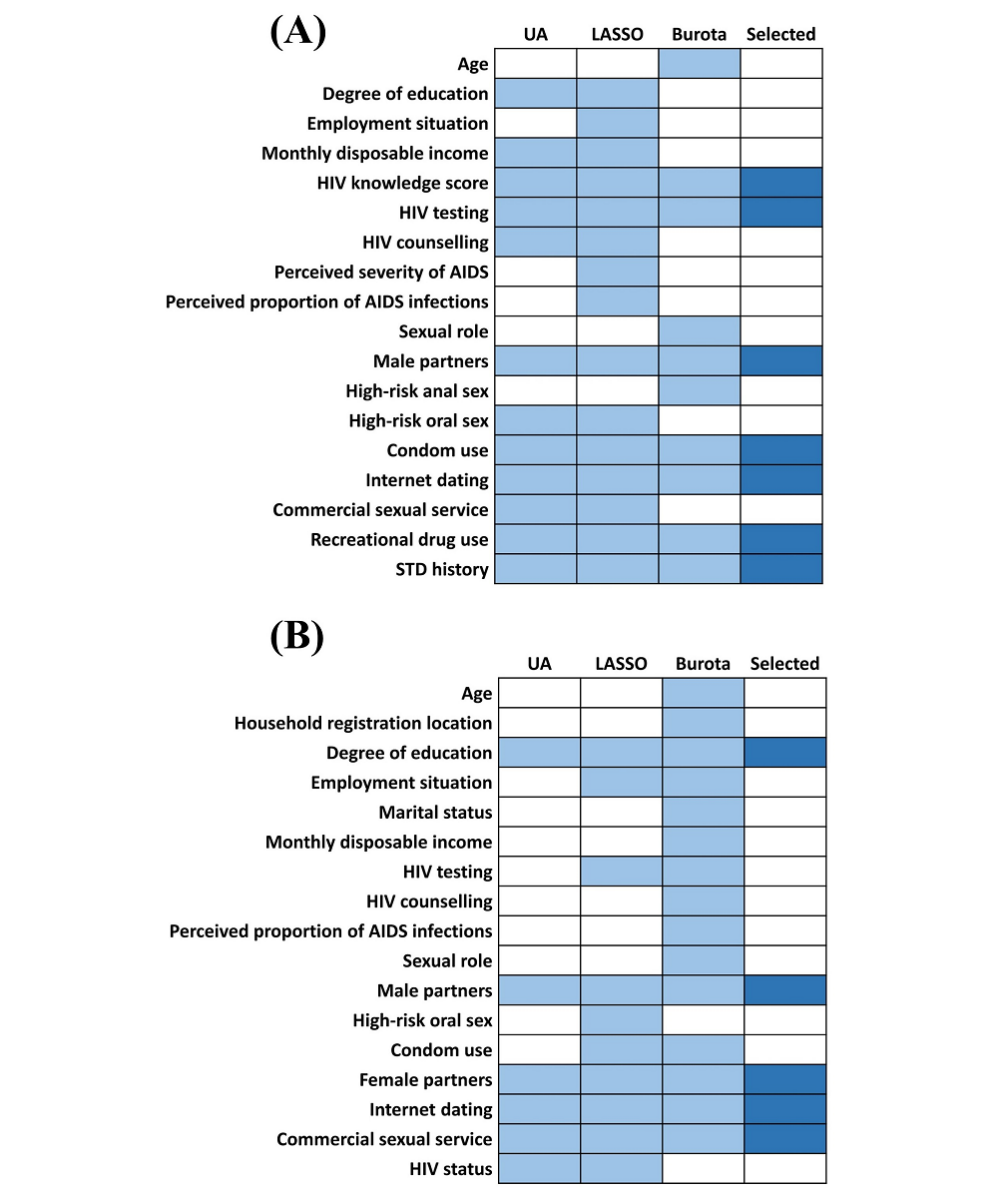
The process of variable screening (A) in the HIV prediction model and (B) in the sexually transmitted disease (STD) prediction model. LASSO: least absolute shrinkage and selection operator; UA: univariate analysis.

LASSO and Boruta were used for feature selection and incorporating factors common to these methods, respectively (refer to Tables S1 and S2 and Figures S2 and S3 in Multimedia Appendix 1 for results of variable screening for each method). On the basis of the results of the statistical analysis, 7 predictors were finally included in the HIV prediction model: HIV knowledge score, HIV testing, number of male partners, condom use, internet dating, recreational drug use, and STD history. A total of 5 predictors were included in the STD prediction model: degree of education, number of male partners, number of female partners, internet dating, and commercial sexual service. The process of variable screening is presented in Figure 2. The results of the selected predictors in the univariate analysis are provided in Table 2.

**Table 2:** Results of selected predictors in HIV and STD^a^ prediction models in univariate analysis (N=1999).

Variables		Participants tested positive for HIV (n=72), n (%)	Participants tested negative for HIV (n=1927), n (%)	*P* value		Participants previously infected with STDs (n=146), n (%)		Participants never infected with STDs (n=1853), n (%)		*P* value	
**Degree of education**					—^b^						.046
	Primary education and lower	—	—			4 (2.7)		38 (2)			
	Junior high school	—	—			16 (10.9)		154 (8.3)			
	Senior high school	—	—			84 (57.5)		911 (49.2)			
	Bachelor’s degree or higher	—	—			42 (28.8)		750 (40.5)			
**HIV knowledge scores**					<.001		—		—		—
	<11	55 (76.4)	1041 (54)								
	≥11	17 (23.6)	886 (46)								
**HIV testing**				<.001		—		—		—	
	No	20 (27.8)	258 (13.4)								
	Yes	52 (72.2)	1669 (86.6)								
**Number of male partners**					.002						.04
	0	5 (77)	293 (15.2)			12 (8.2)		286 (15.4)			
	1	34 (47.2)	1098 (57)			84 (57.5)		1048 (56.6)			
	≥2	33 (45.8)	536 (27.8)			50 (34.2)		519 (28)			
**Condom use**					<.001		—		—		—
	Use every time	31 (43)	1227 (63.7)								
	Use sometimes or occasionally	37 (51.4)	547 (28.4)								
	Never use	4 (5.6)	153 (7.9)								
**Number of female partners**					—						.003
	0	—	—			108 (74)		1571 (84.8)			
	1	—	—			33 (22.6)		246 (13.3)			
	≥2	—	—			5 (3.4)		36 (1.9)			
**Internet dating**					.01						<.001
	No	17 (23.6)	738 (38.3)			33 (22.6)		722 (39)			
	Yes	55 (76.4)	1189 (61.7)			113 (77.4)		1131 (61)			
**Commercial sexual services**					—						.09
	No	—	—			135 (92.5)		1771 (95.6)			
	Yes	—	—			11 (7.5)		82 (4.4)			
**Recreational drug use**					.08^c^		—		—		—
	No	68 (94.4)	1886 (97.9)								
	Yes	4 (5.6)	41 (2.1)								
**STD history**					<.001		—		—		—
	No	58 (80.6)	1795 (93.2)								
	Yes	14 (19.4)	132 (6.8)								

^a^STD: sexually transmitted disease.

^b^Not available.

^c^Fisher exact probability test.

### Model Construction

We randomly divided the data according to a 75% (1499/1999) training set and a 25% (500/1999) testing set, and comparisons of each predictor between the training and testing sets of the HIV and STD prediction models are provided in Tables S3 and S4 in Multimedia Appendix 1. The synthetic minority oversampling technique was used to balance against the dependent variable of the training set. The balanced data were used to predict the risk of HIV infection and STDs using 6 machine learning algorithms, and the predictions were applied to the testing set. The evaluation indicators of the prediction results of each model obtained through the confusion matrix are provided in Table 3 (refer to Figures S4 and S5 in Multimedia Appendix 1 for the confusion matrix of each prediction model).

**Table 3:** The prediction results of each model obtained through the confusion matrix.

Outcomes		AUC^a^ (95% CI)	Accuracy (%)	Balanced accuracy (%)	Sensitivity (%)	Specificity (%)	PPV^b^ (%)	NPV^c^ (%)
**HIV**								
	LR^d^	0.766 (0.641-0.890)	0.874	0.679	0.471	0.889	0.129	0.979
	DT^e^	0.704 (0.545-0.862)	0.786	0.662	0.529	0.795	0.083	0.979
	RF^f^	0.723 (0.591-0.856)	0.824	0.654	0.471	0.836	0.092	0.978
	KNN^g^	0.687 (0.562-0.811)	0.904	0.610	0.294	0.925	0.122	0.973
	LightGBM^h^	0.738 (0.592-0.884)	0.722	0.686	0.647	0.724	0.076	0.983
	XGBoost^i^	0.777 (0.639-0.915)	0.786	0.747	0.706	0.789	0.105	0.987
**STDs** ^j^								
	LR	0.624 (0.536-0.711)	0.582	0.625	0.588	0.582	0.093	0.951
	DT	0.536 (0.442-0.631)	0.592	0.549	0.500	0.599	0.083	0.943
	RF	0.566 (0.473-0.660)	0.586	0.505	0.412	0.599	0.070	0.933
	KNN	0.511 (0.455-0.567)	0.876	0.511	0.088	0.933	0.088	0.933
	LightGBM	0.568 (0.466-0.671)	0.592	0.536	0.471	0.601	0.079	0.940
	XGBoost	0.637 (0.541-0.732)	0.632	0.585	0.618	0.633	0.109	0.958

^a^AUC: area under the curve.

^b^PPV: positive predictive value.

^c^NPV: negative predictive value.

^d^LR: logistic regression.

^e^DT: decision tree.

^f^RF: random forest.

^g^KNN: K-nearest neighbor.

^h^LightGBM: light gradient boosting machine.

^i^XGBoost: extreme gradient boosting.

^j^STD: sexually transmitted disease.

**Figure 3:**
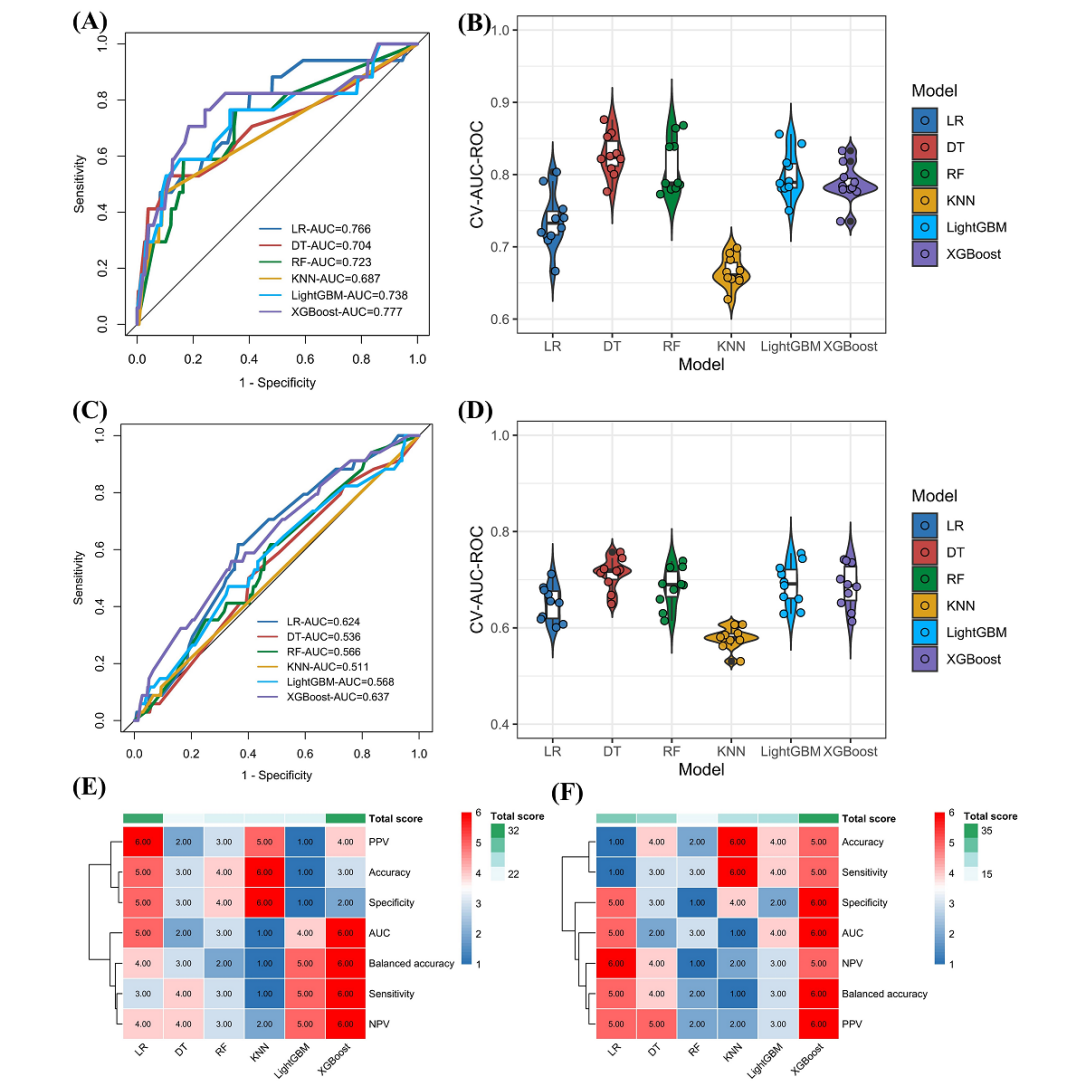
Area under the curve (AUC) values for machine learning models, 10-fold cross-validation, and evaluation indicators: (A) AUC of the HIV prediction model, (B) results of 10-fold cross-validation of the HIV prediction model, (C) AUC of the sexually transmitted disease (STD) prediction model, (D) results of 10-fold cross-validation of the STD prediction model, (E) heat map visualization of the predictive performance of each indicator in HIV prediction model, and (F) heat map visualization of the predictive performance of each indicator in STD prediction model. CV: cross-validation; DT: decision tree; KNN: K-nearest neighbor; LightGBM: light gradient boosting machine; LR: logistic regression; NPV: negative predictive value; PPV: positive predictive value; RF: random forest; ROC: receiver operating characteristic curve; XGBoost: extreme gradient boosting.

The area under the curve (AUC) values of each machine learning model and the results of 10-fold cross-validation are presented in Figures 3A-3D. Figures 3E and 3F show heat maps of the predictive performance data in all models for HIV infection and STD. The results indicated that XGBoost had the highest combined predictive performance scores in the HIV infection and STD prediction models, which were 33 and 39, respectively.

### Interpretability Analysis

**Figure 4:**
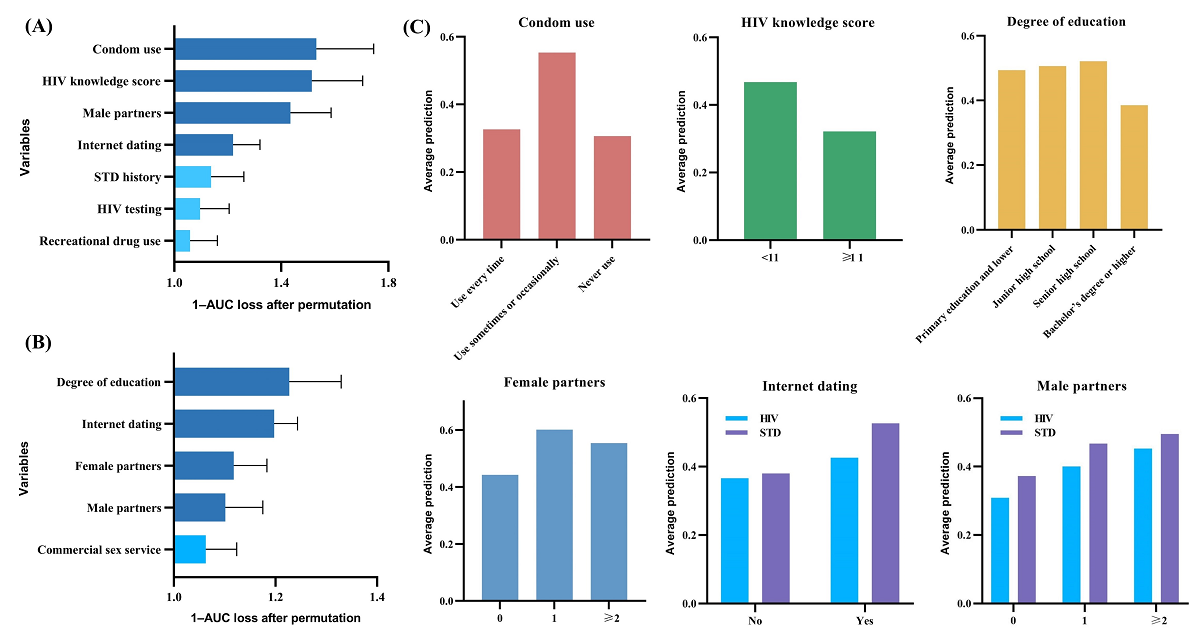
Interpretability analysis of optimal model: (A) importance ranking of variables for HIV prediction model, (B) importance ranking of variables for sexually transmitted disease (STD) prediction model, and (C) partial dependence plots for each variable in the top 4 rankings. AUC: area under the curve; STD: sexually transmitted disease.

The XGBoost algorithm was chosen as the optimal model to rank the importance of all predictors, and the top 4 variables were analyzed for interpretability using the PDPs (Figure 4). According to the results of the study, condom use, HIV knowledge score, number of male partners, and internet dating were the more important variables in the HIV risk prediction model. Degree of education, internet dating, number of female partners, and number of male sexual partners were the more important variables in the STD prediction model. According to the PDPs, MSM who sometimes or occasionally use condoms, have an HIV knowledge score of <11, have ≥2 male partners, and use internet dating reported a higher risk of HIV infection than other MSM. MSM with a low degree of education, those using internet dating, having a female partner, and having ≥2 male partners had a higher risk of STDs than other MSM.

### Risk Stratification

**Figure 5:**
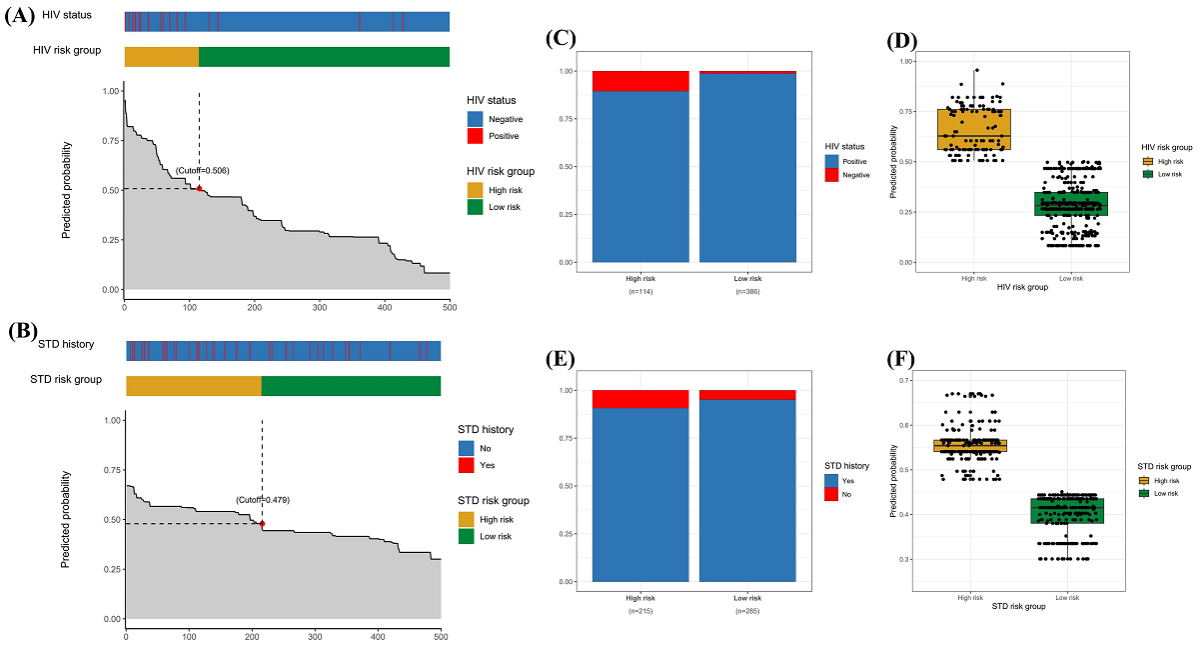
Evaluation of the predicted risk scores of the optimal model in the testing set: (A) distribution of predicted risk scores and risk groups among men who have sex with men (MSM) in HIV prediction model, (B) distribution of predicted risk scores and risk groups among MSM in sexually transmitted disease (STD) prediction model, (C) HIV predicted model’s actual number of infections compared among the risk groups, (D) HIV model’s predicted probability compared among the risk groups, (E) STD predicted model’s actual number of infections compared among the risk groups, and (F) STD model’s predicted probability compared among the risk groups.

On the basis of the cutoff points of the receiver operating characteristic curves of the testing set prediction models, in the HIV prediction model, we defined PRS<0.506 as the low-risk group and PRS ≥0.506 as the high-risk group; in the STD prediction model, we defined PRS<0.479 as the low-risk group and PRS ≥0.479 as the high-risk group. The PRS and risk group distribution of HIV infection and STDs among MSM in the testing set are presented in Figures 5A and 5B. Of the 500 MSM, 386 (77.2%) were in the HIV low-risk group and 114 (22.8%) were in the HIV high-risk group, and 285 (57%) were in the STD low-risk group and 215 (43%) were in the STD high-risk group. The actual number of HIV infections was statistically different in the high- and low-risk groups (*P*<.001; Figure 5C), and the predicted probability of HIV was significantly higher in the high-risk group than in the low-risk group (*P*<.001; Figure 5D). In the high-risk group, the actual number of STDs was higher than in the low-risk group (*P*=.05; Figure 5E), and the predicted probability of STD was significantly higher in the high-risk group than in the low-risk group (*P*<.001; Figure 5F).

### Sensitivity Analysis and Web Applications

Applying the optimal model to an external validation set showed that the model had a fair ability to predict the risk of HIV infection and STDs, with AUC values of 0.627 (95% CI 0.518-0.836) and 0.687 (95% CI 0.528-0.846), respectively. A comparison of differences in demographic characteristics between the external and internal validation sets is provided in Tables S5-S7 in Multimedia Appendix 1. Meanwhile, based on the results of the sensitivity analysis, the optimal model was applied to the MSMM, non-MSMM, MSMO, and MSMW datasets, respectively, and the AUC values ranged from 0.634 to 0.839, indicating that the model was also capable of predicting the risk of HIV infection and STDs in the subgroups (Figure S6 in Multimedia Appendix 1). Finally, the prediction results of the optimal model were displayed in the shiny web applications for probability estimation and interactive computation [29].

## Discussion

### Principal Findings

Our study demonstrated the accuracy of machine learning methods in the prediction of the risk of HIV infection or STDs among MSM. Among the 6 machine learning methods, the XGBoost model outperformed the others, achieving higher AUC values as well as greater accuracy and sensitivity. Furthermore, the most useful predictors were conventional and easy to collect. Optimization-based models were able to stratify the risk of HIV infection and STDs in MSM well. Sensitivity analyses showed that the prediction models for HIV or STD also demonstrated good performance in predicting MSM subgroups with different risks of infection. In addition, the web-based calculators developed in this study provided a valuable tool for clinicians and MSM to make more informed behavioral decisions. These data could guide prevention and intervention programs, allowing users to tailor interventions to the risk profile of the individual. The routine collection of data, the accuracy of the prediction models, and the convenience of the web applications suggested that machine learning methods may be of value in HIV infection or STD risk assessment services.

Machine learning methods offer flexibility and scalability, making them suitable for a variety of tasks, such as risk stratification, especially when analyzing big data [30]. Risk prediction models are widely used in clinical scenarios, and clinicians may be able to use clinical prediction models to quantify predictions and more intuitively present their risk of infection and intervention options to the MSM population, potentially moderating the information asymmetry between physicians and patients to some extent. In addition, risk prediction models can play a role in the tertiary prevention system of diseases. For example, according to the individual characteristics of high-risk groups, the risk of potential future infections can be quantified, providing a more intuitive and scientific reference for health education and behavioral interventions, and noninvasive, cost-effective, and convenient indicators can be used for risk screening, thus realizing secondary prevention of “early detection, early diagnosis, and early treatment.” Clinicians can more accurately screen appropriate study participants, and government departments and health management decision makers can better manage the quality of health care and rationally allocate health care resources. Meanwhile, for MSM, web applications enable self-assessment of infection risk anytime and anywhere based on their individual situations, which is conducive to protecting their privacy and strengthening awareness of self-management and self-monitoring among high-risk groups.

In our study, for the MSM population in Western China, our prediction results were consistent with previous prediction studies and better predicted the risk of infection. A study conducted to predict future occurrence of syphilis in a high-risk cohort of MSM and transgender women in Peru reported an AUC value of 0.690 [10]. In HIV diagnosis studies of MSM in Australia, the AUC value of their prediction models ranged from 0.698 to 0.763, and for the diagnosis of other STDs, the AUC value ranged from 0.632 to 0.858 [31]. However, the STD prediction model in this study had a slightly lower AUC value (0.777 vs 0.637) compared to the HIV prediction model, which may be related to the way the dependent variables were collected. We objectively measured the HIV infection status of MSM through HIV antigen-antibody testing, whereas the STD history of MSM was obtained through subjective self-reports. MSM may have chosen to conceal their infection history due to social desirability bias, leading to an underestimation of outcomes [32]. Although subjective measurements may lead to a reduction in the efficacy of prediction models, their predictions were generally reliable. At the same time, according to the results of the HIV prediction model, STD history was also an important factor in HIV infection, which was consistent with the results of previous studies [10,33]. In our study, we developed separate prediction models for HIV infection and STDs, designed to function as a *2-step* risk assessment tool. The first step evaluates the risk of STDs in MSM, followed by the second step, which assesses the risk of HIV, offering more comprehensive information for HIV risk prediction.

Our results showed that the XGBoost algorithm performed optimally in predicting the risk of HIV infection and STDs in the MSM population in Western China. XGBoost is an enhanced version of the gradient boosting machine. The optimal performance of XGBoost has been reported in studies predicting HIV infection in Zimbabwe from 2005 to 2015 and in studies predicting timely clinic attendance and acceptance of HIV infection or STD testing [34,35]. The XGBoost algorithm incorporates regular terms to control the complexity of the model, which can effectively prevent overfitting. In addition, XGBoost modeling has been shown to gain signal from missingness without resorting to imputation techniques [36]. In summary, in predicting the risk of HIV infection and STDs in the MSM population in Western China, XGBoost has reported the best prediction performance in terms of discriminative ability and clinical application. XGBoost can be used as a prioritized algorithm in future risk prediction tools.

According to the interpretability analysis of the prediction model, condom use was one of the important factors in predicting the risk of HIV infection in the MSM population, which was also consistent with the results of previous studies [31]. High-risk sexual behaviors, such as unprotected sex and inconsistent condom use, may accelerate HIV transmission between MSM [37,38]. According to our findings, MSM who sometimes or occasionally used condoms were predicted to have a higher mean probability of infection than MSM who used condoms every time. However, the probability of infection among MSM who never used condoms was not high, and we believe that it may be related to the social desirability bias of MSM, which made them reluctant or afraid to admit that they did not use condoms, leading to an underestimation of this outcome.

In addition, our findings showed that MSM who found sexual partners through the internet (ie, internet dating) had a higher risk of both HIV infection and STD than MSM who did not. Considering that individuals have free access to explicit sexual content on the internet, coupled with the increased risk of MSM infection, the role of the internet in sexual partner encounters and its correlation with risky sexual behavior has become an important topic [39]. The internet was known to be a popular place for MSM to find sexual partners; for example, previous studies found that 40% of MSM used the internet to find sexual partners [40,41]. Previous studies have shown that MSM who use internet dating are more likely to engage in psychotropic drug use and unprotected intercourse compared to other MSM [42]. This result was consistent with our findings. It is evident that targeted measures should be taken to improve their attitudes and perceptions and reduce their risk of infection.

A study in the United States showed that MSM with multiple partners are at higher risk of HIV infection [43]. This finding was confirmed by 2 national cross-sectional surveys conducted in Brazil in 2009 and 2016 [44]. In addition, previous studies in China have found that MSM with multiple sexual partners and those having unprotected sex were at increased risk of HIV infection [26]. These were consistent with our findings. It is clear that MSM with multiple sex partners should be given timely attention and education to reduce their risk of infection. Moreover, our prediction model screened for variables such as HIV knowledge score, degree of education, and HIV testing. All of these variables were significant predictors of infection risk, and interventions targeting these variables were also important for HIV infection and STD prevention in the MSM population.

### Limitations

In summary, we have developed risk prediction tools that can be widely used in real-life settings. All predictors are self-reported risks, and a user-friendly, web-based self-assessment tool has the potential to help MSM identify their risk of HIV infection or STDs in a nonclinical setting, which enables them to determine their risk of infection and seek timely medical help. Meanwhile, in clinical scenarios, these tools will help clinicians stratify high-risk individuals and be more attentive to individuals who are potentially at high risk of infection. However, there were some limitations in our study. First, we recruited participants using nonprobability sampling, which may have led to bias and limited the generalizability of this study (refer to Multimedia Appendix 1 for the evaluation of the bias in the data relative to the population). In addition, our study relied on voluntary participation by MSM, and the characteristics of participants may have differed from those of nonparticipants, leading to self-selection bias. The validity of risk prediction models depended on the accuracy of self-reported information, which was influenced by MSM recall bias and social desirability bias. In future research on MSM, some accurate and reliable information gathering tools are necessary, for example, computer-assisted self-interview [31]. Our study revealed significant spatial heterogeneity in the demographic characteristics and the risk of HIV infection or STDs among the MSM population in Western China. This heterogeneity reflects regional differences in population structure, socioeconomic conditions, behavioral patterns, and access to health care services. Considering the epidemiological context, these findings suggest the need to develop tailored prevention and control strategies based on regional characteristics to effectively reduce the risk of HIV and STD transmission in various areas (Figure S7 and Table S8 in Multimedia Appendix 1). Second, the sample size of our study may be smaller than that of other large-scale national survey studies. As the study focused the MSM population, conducting a large-scale national survey was difficult. However, the results of risk prediction models and sensitivity analyses showed that, with the current sample size, the predictions were considerable and could be used in real-world applications. Third, we only used 6 machine learning methods for risk prediction, and others may be explored as part of future research. Fourth, although the optimal prediction model performed moderately well in the MSM in Sichuan (ie, the external validation set), our analysis, presented in Tables S5-S7 in Multimedia Appendix 1, revealed significant differences in demographic variables between the external and internal validation sets. These discrepancies may have contributed to inaccurate predictions, but they also highlighted the authenticity of the external data used to test the risk prediction model, as such differences in the external validation dataset were unavoidable. Future attempts should be made to apply external validation to data from multiple regions to demonstrate the robustness of the prediction model. Finally, our study represented risk projections of outcomes and characteristics from cross-sectional surveys rather than prospective cohort projects, and longitudinal projections will be part of future surveys. As the models were constructed only from Western China, further exploration and validation were needed to generalize the optimal model to the whole country to make it universally applicable.

### Conclusions

Our results showed that the machine learning approach demonstrated good performance in predicting the risk of HIV infection or STDs in the MSM population in Western China. The method could be used for the risk stratification of MSM, which would help clinicians and MSM to conduct risk assessment and intervene and prevent the risk in a timely manner. At the same time, user-friendly and easily operated web applications have important public health implications in risk monitoring among the MSM population and can be widely used in reality.

## Data Availability

The datasets generated and analyzed during this study are available from the corresponding author on reasonable request.

## Appendix

Multimedia Appendix 1 Supplementary analyses, figures, and tables.

## Abbreviations

AUC: area under the curve
LASSO: least absolute shrinkage and selection operator
MSM: men who have sex with men
MSMM: men who have sex with multiple men
MSMO: men who have sex with men only
MSMW: men who have sex with men and women
PDP: partial dependence plot
PRS: predicted risk score
STD: sexually transmitted disease
XGBoost: extreme gradient boosting

## References

[1] (2023). The path that ends AIDS: 2023 UNAIDS global AIDS update. Joint United Nations Program on HIV/AIDS.

[2] Dong MJ, Peng B, Liu ZF, Ye QN, Liu H, Lu XL, Zhang B, Chen JJ (2019). The prevalence of HIV among MSM in China: a large-scale systematic analysis. BMC Infect Dis.

[3] Lu R, Zhang X, Zhou C, Zhang W, Ouyang L, Xing H, Shao Y, Ruan Y, Wu G (2020). Trends of human immunodeficiency virus, syphilis, and hepatitis c infections among men who have sex with men in Chongqing, China: a serial cross-sectional survey from 2011 to 2018. Sexual Trans Dis.

[4] Chow EP, Grulich AE, Fairley CK (2019). Epidemiology and prevention of sexually transmitted infections in men who have sex with men at risk of HIV. Lancet HIV.

[5] Fan L, Yu A, Zhang D, Wang Z, Ma P (2021). Consequences of HIV/syphilis co-infection on HIV viral load and immune response to antiretroviral therapy. Infect Drug Resist.

[6] Yang LG, Zhang XH, Zhao PZ, Chen ZY, Ke WJ, Ren XQ, Wang LY, Chen WY, Tucker JD (2018). Gonorrhea and chlamydia prevalence in different anatomical sites among men who have sex with men: a cross-sectional study in Guangzhou, China. BMC Infect Dis.

[7] Lin XX, Meng SY, Ke WJ, Zhang XH, Wang LY, Liao YY, Liu H, Zhao PZ, Liang CM, Chen HR, Long HY, Yang B, Yang LG (2022). Community engagement on-site rapid test for chlamydia and gonorrhea among men who have sex with men: a pioneering study in Guangzhou, China. BMC Public Health.

[8] Marchese V, Tiecco G, Storti S, Degli Antoni M, Calza S, Gulletta M, Viola F, Focà E, Matteelli A, Castelli F, Quiros-Roldan E (2022). Syphilis infections, reinfections and serological response in a large Italian sexually transmitted disease centre: a monocentric retrospective study. J Clin Med.

[9] Hoenigl M, Weibel N, Mehta SR, Anderson CM, Jenks J, Green N, Gianella S, Smith DM, Little SJ (2015). Development and validation of the San Diego Early Test Score to predict acute and early HIV infection risk in men who have sex with men. Clin Infect Dis.

[10] Allan-Blitz LT, Konda KA, Vargas SK, Wang X, Segura ER, Fazio BM, Calvo GM, Caceres CF, Klausner JD (2018). The development of an online risk calculator for the prediction of future syphilis among a high-risk cohort of men who have sex with men and transgender women in Lima, Peru. Sex Health.

[11] Wand H, Reddy T, Naidoo S, Moonsamy S, Siva S, Morar NS, Ramjee G (2018). A simple risk prediction algorithm for HIV transmission: results from HIV prevention trials in KwaZulu Natal, South Africa (2002-2012). AIDS Behav.

[12] He J, Li J, Jiang S, Cheng W, Jiang J, Xu Y, Yang J, Zhou X, Chai C, Wu C (2022). Application of machine learning algorithms in predicting HIV infection among men who have sex with men: model development and validation. Front Public Health.

[13] Ji W, Wang C, Chen H, Liang Y, Wang S (2023). Predicting post-stroke cognitive impairment using machine learning: a prospective cohort study. J Stroke Cerebrovasc Dis.

[14] Yi F, Yang H, Chen D, Qin Y, Han H, Cui J, Bai W, Ma Y, Zhang R, Yu H (2023). XGBoost-SHAP-based interpretable diagnostic framework for Alzheimer's disease. BMC Med Inform Decis Mak.

[15] Wang X, Qiao Y, Cui Y, Ren H, Zhao Y, Linghu L, Ren J, Zhao Z, Chen L, Qiu L (2023). An explainable artificial intelligence framework for risk prediction of COPD in smokers. BMC Public Health.

[16] Lundberg SM, Nair B, Vavilala MS, Horibe M, Eisses MJ, Adams T, Liston DE, Low DK, Newman SF, Kim J, Lee SI (2018). Explainable machine-learning predictions for the prevention of hypoxaemia during surgery. Nat Biomed Eng.

[17] Cabitza F, Rasoini R, Gensini GF (2017). Unintended consequences of machine learning in medicine. JAMA.

[18] Leme DE, de Oliveira C (2023). Machine learning models to predict future frailty in community-dwelling middle-aged and older adults: the ELSA cohort study. J Gerontol A Biol Sci Med Sci.

[19] Sampa MB, Biswas T, Rahman MS, Aziz NH, Hossain MN, Aziz NA (2023). A machine learning web app to predict diabetic blood glucose based on a basic noninvasive health checkup, sociodemographic characteristics, and dietary information: case study. JMIR Diabetes.

[20] Karabacak M, Jagtiani P, Carrasquilla A, Germano IM, Margetis K (2023). Prognosis individualized: survival predictions for WHO grade II and III gliomas with a machine learning-based web application. NPJ Digit Med.

[21] Lei M, Wu B, Zhang Z, Qin Y, Cao X, Cao Y, Liu B, Su X, Liu Y (2023). A web-based calculator to predict early death among patients with bone metastasis using machine learning techniques: development and validation study. J Med Internet Res.

[22] Lin B, Liu J, Ma Y, Zhong X (2022). Factors influencing HIV testing and counselling services among men who have sex with men in Western China: a cross-sectional study based on Andersen's Behavioral Model. Environ Health Prev Med.

[23] Cui Y, Shi X, Wang S, Qin Y, Wang B, Che X, Lei M (2022). Machine learning approaches for prediction of early death among lung cancer patients with bone metastases using routine clinical characteristics: an analysis of 19,887 patients. Front Public Health.

[24] Gao Y, Xin L, Lin H, Yao B, Zhang T, Zhou AJ, Huang S, Wang JH, Feng YD, Yao SH, Guo Y, Dang T, Meng XM, Yang ZZ, Jia WQ, Pang HF, Tian XJ, Deng B, Wang JP, Fan WC, Wang J, Shi LH, Yang GY, Sun C, Wang W, Zang JC, Li SY, Shi RH, Li ZS, Wang LW (2023). Machine learning-based automated sponge cytology for screening of oesophageal squamous cell carcinoma and adenocarcinoma of the oesophagogastric junction: a nationwide, multicohort, prospective study. Lancet Gastroenterol Hepatol.

[25] Hu Y, Zhong XN, Peng B, Zhang Y, Liang H, Dai JH, Zhang J, Zhong XH, Huang AL (2019). Comparison of depression and anxiety between HIV-negative men who have sex with men and women (MSMW) and men who have sex with men only (MSMO): a cross-sectional study in Western China. BMJ Open.

[26] Liu J, Deng R, Lin B, Pan H, Gao Y, Dai J, Liang H, Huang A, Zhong X (2021). Risk management on pre-exposure prophylaxis adherence of men who have sex with multiple men: a multicenter prospective cohort study. Risk Manag Healthc Policy.

[27] Ramakrishnan L, Ramanathan S, Chakrapani V, Goswami P, Deshpande S, Yadav D, Sen S, George B, Paranjape R (2015). Comparison of sexual risk, HIV/STI prevalence and intervention exposure among men who have sex with men and women (MSMW) and men who have sex with men only (MSMO) in India: implications for HIV prevention. AIDS Behav.

[28] Yin L, Zhao Y, Peratikos MB, Song L, Zhang X, Xin R, Sun Z, Xu Y, Zhang L, Hu Y, Hao C, Ruan Y, Shao Y, Vermund SH, Qian HZ (2018). Risk prediction score for HIV infection: development and internal validation with cross-sectional data from men who have sex with men in China. AIDS Behav.

[29] A Shiny web application for predicting HIV among MSM based on machine learning. Shiny App.

[30] Ngiam KY, Khor IW (2019). Big data and machine learning algorithms for health-care delivery. Lancet Oncol.

[31] Bao Y, Medland NA, Fairley CK, Wu J, Shang X, Chow EP, Xu X, Ge Z, Zhuang X, Zhang L (2021). Predicting the diagnosis of HIV and sexually transmitted infections among men who have sex with men using machine learning approaches. J Infect.

[32] Firkey MK, Buckheit KA, Mitzel LD, Maisto SA, Palfai T, Vanable P (2020). Sexual risk and social desirability among Black and White men who have sex with men. J Black Sexuality Relatsh.

[33] Solomon MM, Mayer KH, Glidden DV, Liu AY, McMahan VM, Guanira JV, Chariyalertsak S, Fernandez T, Grant RM (2014). Syphilis predicts HIV incidence among men and transgender women who have sex with men in a preexposure prophylaxis trial. Clin Infect Dis.

[34] Birri Makota RB, Musenge E (2023). Predicting HIV infection in the decade (2005-2015) pre-COVID-19 in Zimbabwe: a supervised classification-based machine learning approach. PLOS Digit Health.

[35] Xu X, Fairley CK, Chow EP, Lee D, Aung ET, Zhang L, Ong JJ (2022). Using machine learning approaches to predict timely clinic attendance and the uptake of HIV/STI testing post clinic reminder messages. Sci Rep.

[36] Zhang X, Yan C, Gao C, Malin BA, Chen Y (2020). Predicting missing values in medical data via XGBoost regression. J Healthc Inform Res.

[37] Traeger MW, Schroeder SE, Wright EJ, Hellard ME, Cornelisse VJ, Doyle JS, Stoové MA (2018). Effects of pre-exposure prophylaxis for the prevention of human immunodeficiency virus infection on sexual risk behavior in men who have sex with men: a systematic review and meta-analysis. Clin Infect Dis.

[38] Cao Z, Chen J, Lin B, Zhang C, Zhong X (2023). Factors influencing intention on condom use during sexual intercourse with regular female partners among men who have sex with men in Western China: a structural equation modeling analysis. Sexual Trans Dis.

[39] Xu J, Luo Y, Dong H, Zhao G (2022). The effects of internet exposure on sexual risk behavior among sexually experienced male college students in China: cross-sectional study. JMIR Public Health Surveill.

[40] Phillips G 2nd, Magnus M, Kuo I, Rawls A, Peterson J, Jia Y, Opoku J, Greenberg AE (2014). Use of geosocial networking (GSN) mobile phone applications to find men for sex by men who have sex with men (MSM) in Washington, DC. AIDS Behav.

[41] Holloway IW, Pulsipher CA, Gibbs J, Barman-Adhikari A, Rice E (2015). Network influences on the sexual risk behaviors of gay, bisexual and other men who have sex with men using geosocial networking applications. AIDS Behav.

[42] Zhao P, Tang S, Wang C, Zhang Y, Best J, Tangthanasup TM, Huang S, Yang B, Wei C, Tucker JD, Tang W (2017). Recreational drug use among Chinese MSM and transgender individuals: results from a national online cross-sectional study. PLoS One.

[43] Chittamuru D, Icard LD, Jemmott JB 3rd, O'Leary A (2018). Prospective predictors of multiple sexual partners among African American men who have sex with men. Arch Sex Behav.

[44] Guimarães MD, Kendall C, Magno L, Rocha GM, Knauth DR, Leal AF, Dourado I, Veras MA, Brito AM, Kerr LR (2018). Comparing HIV risk-related behaviors between 2 RDS national samples of MSM in Brazil, 2009 and 2016. Medicine (Baltimore).

