# Supplementary material for: Predicting the Risk of HIV Infection and Sexually Transmitted Diseases Among Men Who Have Sex With Men: Cross-Sectional Study Using Multiple Machine Learning Approaches
Source: J Med Internet Res. 2025 Feb 20;27:e59101. doi: 10.2196/59101 (PMC11888048; doi:10.2196/59101)
Supplement: Multimedia Appendix 1 [file jmir_v27i1e59101_app1.docx]

**Screening process flowcharts, result tables and figures**

**Multimedia Appendix 1**

**1. A flowchart of the participant screening process**

A flowchart of the screening process for the study participants was shown in **Figure S1**. According to Supplementary Figure 1, we recruited a total of 2346 MSM in western China between 2013 and 2023, and a total of 1999 MSM were included in the analyses after exclusion of those who did not meet the requirements. The basic demographic information of the 1999 MSM is shown in **Table 1**. The highest percentage of MSM in the recruited population was 25-35 years old at 36.62%, and recruitment was generally among younger MSM. The urban population was relatively high (71.54%), mostly Han ethnicity (91.60%), with a high level of education. The majority of MSM were employed (79.59%), most self-reported as unmarried (84.29%), and fewer were in higher income groups (5.00%).


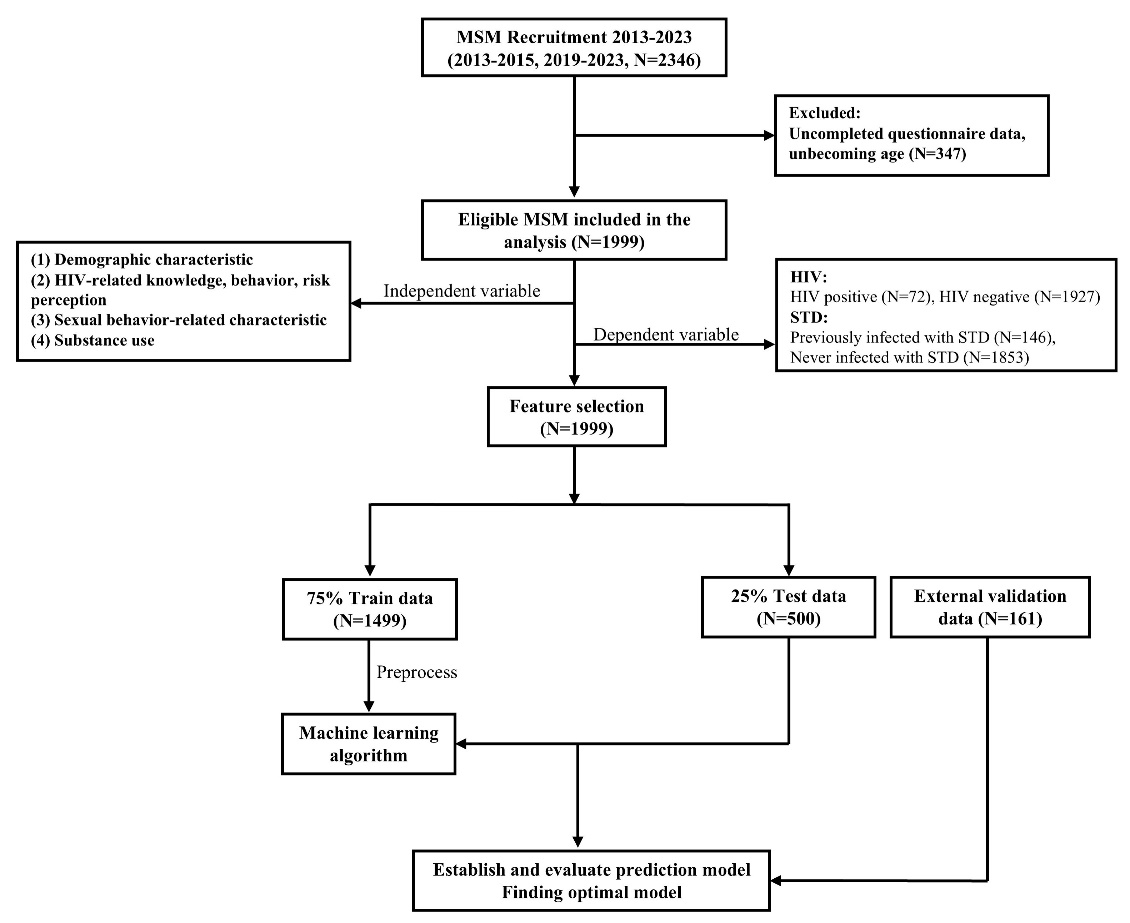


**Figure S1. A flowchart of the participant screening process**

MSM: Men who have sex with men; HIV: Human immunodeficiency virus; STD: Sexually transmitted diseases.

**2. Results of variable screening**

In our study, we employed three methods, UA, LASSO, and Boruta, respectively, for variable selection, and ultimately included factors common to all these methods. The results of the univariate analysis in the risk prediction model for HIV were shown in **Table S1**. Based on the results of the univariate analysis, variables with p-value<0.1 were selected, degree of education (p=0.0222), monthly disposable income (p=0.0197), HIV knowledge score (p=0.0002), HIV testing (p=0.0005), HIV counseling (p=0.0044), number of male partners (p=0.0022), high-risk oral sex (p=0.0025), condom use (p=0.0001), Internet dating (p=0.0116), commercial sexual services (p=0.0468), recreational drug use (p=0.0759), and STD history (p<0.0001) were the 12 variables selected.

**Table S1. Univariate analysis in the risk prediction model for HIV**

| Variables | HIV Positive  (N=72) | | HIV Negative  (N=1927) | | p-value |
| --- | --- | --- | --- | --- | --- |
|  | N | % | N | % |  |
| Age |  |  |  |  | 0.2418 |
| 18-25 | 1 | 1.39 | 146 | 7.58 |  |
| 25-35 | 26 | 36.11 | 706 | 36.64 |  |
| 35-45 | 28 | 38.89 | 661 | 34.30 |  |
| ≥45 | 17 | 23.61 | 414 | 21.48 |  |
| Household registration location |  |  |  |  | 0.6888 |
| Urban | 50 | 69.44 | 1380 | 71.61 |  |
| Rural | 22 | 30.56 | 547 | 28.39 |  |
| Ethnicity |  |  |  |  | 0.3749 |
| Han | 68 | 94.44 | 1763 | 91.49 |  |
| Ethnic minorities | 4 | 5.56 | 164 | 8.51 |  |
| Degree of education |  |  |  |  | 0.0222 |
| Primary education and lower | 2 | 2.78 | 40 | 2.08 |  |
| Junior high school | 7 | 9.72 | 163 | 8.46 |  |
| Senior high school | 47 | 65.28 | 948 | 49.20 |  |
| Bachelor’s degree or higher | 16 | 22.22 | 776 | 40.27 |  |
| Employment situation |  |  |  |  | 0.2859 |
| Unemployed | 9 | 12.50 | 168 | 8.72 |  |
| Employed | 58 | 80.56 | 1533 | 79.55 |  |
| Student | 5 | 6.94 | 226 | 11.73 |  |
| Marital status |  |  |  |  | 0.8199 |
| Unmarried | 60 | 83.33 | 1625 | 84.33 |  |
| Married | 12 | 16.67 | 302 | 15.67 |  |
| Monthly disposable income |  |  |  |  | 0.0197 |
| 1000-3000 RMB | 40 | 55.55 | 771 | 40.01 |  |
| 3000-10000 RMB | 31 | 43.06 | 1057 | 54.85 |  |
| ≥10000 RMB | 1 | 1.39 | 99 | 5.14 |  |
| HIV knowledge scores |  |  |  |  | 0.0002 |
| <11 | 55 | 76.39 | 1041 | 54.02 |  |
| ≥11 | 17 | 23.61 | 886 | 45.98 |  |
| HIV testing |  |  |  |  | 0.0005 |
| No | 20 | 27.78 | 258 | 13.39 |  |
| Yes | 52 | 72.22 | 1669 | 86.61 |  |
| HIV counseling |  |  |  |  | 0.0044 |
| No | 34 | 47.22 | 603 | 31.29 |  |
| Yes | 38 | 52.78 | 1324 | 68.71 |  |
| HIV risk perception: perceived severity of AIDS |  |  |  |  | 0.1932 |
| Low level | 0 | 0.00 | 15 | 0.78 |  |
| Middle level | 2 | 2.78 | 155 | 8.04 |  |
| High level | 70 | 97.22 | 1757 | 91.18 |  |
| HIV risk perception: perceived proportion of AIDS infections |  |  |  |  | 0.3453 |
| Low level | 8 | 11.12 | 188 | 9.76 |  |
| Middle level | 14 | 19.44 | 524 | 27.19 |  |
| High level | 50 | 69.44 | 1215 | 63.05 |  |
| HIV risk perception: perceived threat of AIDS |  |  |  |  | 0.8071 |
| Low level | 5 | 6.94 | 176 | 9.13 |  |
| Middle level | 9 | 12.50 | 247 | 12.82 |  |
| High level | 58 | 80.56 | 1504 | 78.05 |  |
| Sexual role |  |  |  |  | 0.8380 |
| Receiver | 20 | 27.78 | 485 | 25.17 |  |
| Both | 18 | 25.00 | 468 | 24.29 |  |
| Inserter | 34 | 47.22 | 974 | 50.54 |  |
| Number of male partners |  |  |  |  | 0.0022 |
| 0 | 5 | 6.95 | 293 | 15.20 |  |
| 1 | 34 | 47.22 | 1098 | 56.98 |  |
| ≥2 | 33 | 45.83 | 536 | 27.82 |  |
| High-risk anal sex (Condom use during anal sex) |  |  |  |  | 0.3958 |
| No | 63 | 87.50 | 1744 | 90.50 |  |
| Yes | 9 | 12.50 | 183 | 9.50 |  |
| High-risk oral sex (Condom use during oral sex) |  |  |  |  | 0.0025 |
| No | 18 | 25.00 | 827 | 42.92 |  |
| Yes | 54 | 75.00 | 1100 | 57.08 |  |
| Condom use |  |  |  |  | 0.0001 |
| Use every time | 31 | 43.05 | 1227 | 63.67 |  |
| Use sometimes or occasionally | 37 | 51.39 | 547 | 28.39 |  |
| Never use | 4 | 5.56 | 153 | 7.94 |  |
| Number of female partners |  |  |  |  | 0.5589 |
| 0 | 58 | 80.55 | 1621 | 84.12 |  |
| 1 | 13 | 18.06 | 266 | 13.80 |  |
| ≥2 | 1 | 1.39 | 40 | 2.08 |  |
| Internet dating |  |  |  |  | 0.0116 |
| No | 17 | 23.61 | 738 | 38.30 |  |
| Yes | 55 | 76.39 | 1189 | 61.70 |  |
| Alcohol use |  |  |  |  | 0.4980 |
| Every day | 3 | 4.17 | 87 | 4.51 |  |
| At least 3 times per week | 10 | 13.89 | 154 | 7.99 |  |
| At least 1 time per week | 10 | 13.89 | 273 | 14.17 |  |
| Less than 1 time per week | 24 | 33.33 | 732 | 37.99 |  |
| Never drink | 25 | 34.72 | 681 | 35.34 |  |
| Commercial sexual services |  |  |  |  | 0.0468^ |
| No | 65 | 90.28 | 1841 | 95.54 |  |
| Yes | 7 | 9.72 | 86 | 4.46 |  |
| Recreational drug use |  |  |  |  | 0.0759^ |
| No | 68 | 94.44 | 1886 | 97.87 |  |
| Yes | 4 | 5.56 | 41 | 2.13 |  |
| STD history |  |  |  |  | <0.0001 |
| No | 58 | 80.56 | 1795 | 93.15 |  |
| Yes | 14 | 19.44 | 132 | 6.85 |  |

HIV: Human immunodeficiency virus; RMB: the legal tender of China, the unit is Yuan; AIDS: Acquired immune deficiency syndrome; STD: Sexually transmitted diseases.

^：Fisher exact probability test.

In the risk prediction model for STD, the results of the univariate analysis were shown in **Table S2**. Based on the results of the univariate analysis, variables with p-value<0.1 were selected. The six variables of degree of education (p=0.0458), number of male partners (p=0.0369), number of female partners (p=0.0028), Internet dating (p<0.0001), commercial sexual services (p=0.0859), and HIV status (p<0.0001) were selected.

**Table S2. Univariate analysis in the risk prediction model for STD**

| Variables | Previously infected with STD  (N=146) | | Never infected with STD  (N=1853) | | p-value |
| --- | --- | --- | --- | --- | --- |
|  | N | % | N | % |  |
| Age |  |  |  |  | 0.1097 |
| 18-25 | 10 | 6.85 | 137 | 7.39 |  |
| 25-35 | 56 | 38.36 | 676 | 36.48 |  |
| 35-45 | 39 | 26.71 | 650 | 35.08 |  |
| ≥45 | 41 | 28.08 | 390 | 21.05 |  |
| Household registration location |  |  |  |  | 0.1563 |
| Urban | 97 | 66.44 | 1333 | 71.94 |  |
| Rural | 49 | 33.56 | 520 | 28.06 |  |
| Ethnicity |  |  |  |  | 0.5920 |
| Han | 132 | 90.41 | 1699 | 91.69 |  |
| Ethnic minorities | 14 | 9.59 | 154 | 8.31 |  |
| Degree of education |  |  |  |  | 0.0458 |
| Primary education and lower | 4 | 2.74 | 38 | 2.05 |  |
| Junior high school | 16 | 10.96 | 154 | 8.31 |  |
| Senior high school | 84 | 57.53 | 911 | 49.16 |  |
| Bachelor’s degree or higher | 42 | 28.77 | 750 | 40.47 |  |
| Employment situation |  |  |  |  | 0.1132 |
| Unemployed | 18 | 12.33 | 159 | 8.58 |  |
| Employed | 117 | 80.14 | 1474 | 79.55 |  |
| Student | 11 | 7.53 | 220 | 11.87 |  |
| Marital status |  |  |  |  | 0.3369 |
| Unmarried | 119 | 81.51 | 1566 | 84.51 |  |
| Married | 27 | 18.49 | 287 | 15.49 |  |
| Monthly disposable income |  |  |  |  | 0.8295 |
| 1000-3000 RMB | 62 | 42.47 | 749 | 40.42 |  |
| 3000-10000 RMB | 76 | 52.05 | 1012 | 54.62 |  |
| ≥10000 RMB | 8 | 5.48 | 92 | 4.96 |  |
| HIV knowledge scores |  |  |  |  | 0.6101 |
| <11 | 83 | 56.85 | 1013 | 54.67 |  |
| ≥11 | 63 | 43.15 | 840 | 45.33 |  |
| HIV testing |  |  |  |  | 0.1173 |
| No | 14 | 9.59 | 264 | 14.25 |  |
| Yes | 132 | 90.41 | 1589 | 85.75 |  |
| HIV counseling |  |  |  |  | 0.7786 |
| No | 45 | 30.82 | 592 | 31.95 |  |
| Yes | 101 | 69.18 | 1261 | 68.05 |  |
| HIV risk perception: perceived severity of AIDS |  |  |  |  | 0.4954 |
| Low level | 0 | 0.00 | 15 | 0.81 |  |
| Middle level | 13 | 8.90 | 144 | 7.77 |  |
| High level | 133 | 91.10 | 1694 | 91.42 |  |
| HIV risk perception: perceived proportion of AIDS infections |  |  |  |  | 0.2709 |
| Low level | 16 | 10.96 | 180 | 9.71 |  |
| Middle level | 31 | 21.23 | 507 | 27.36 |  |
| High level | 99 | 67.81 | 1166 | 62.93 |  |
| HIV risk perception: perceived threat of AIDS |  |  |  |  | 0.7772 |
| Low level | 13 | 8.90 | 168 | 9.07 |  |
| Middle level | 16 | 10.96 | 240 | 12.95 |  |
| High level | 117 | 80.14 | 1445 | 77.98 |  |
| Sexual role |  |  |  |  | 0.3527 |
| Receiver | 33 | 22.60 | 472 | 25.47 |  |
| Both | 31 | 21.24 | 455 | 24.56 |  |
| Inserter | 82 | 56.16 | 926 | 49.97 |  |
| Number of male partners |  |  |  |  | 0.0369 |
| 0 | 12 | 8.22 | 286 | 15.43 |  |
| 1 | 84 | 57.53 | 1048 | 56.56 |  |
| ≥2 | 50 | 34.25 | 519 | 28.01 |  |
| High-risk anal sex (Condom use during anal sex) |  |  |  |  | 0.2405 |
| No | 136 | 93.15 | 1671 | 90.18 |  |
| Yes | 10 | 6.85 | 182 | 9.82 |  |
| High-risk oral sex (Condom use during oral sex) |  |  |  |  | 0.4560 |
| No | 66 | 45.21 | 779 | 42.04 |  |
| Yes | 80 | 54.79 | 1074 | 57.96 |  |
| Condom use |  |  |  |  | 0.1081 |
| Use every time | 103 | 70.55 | 1155 | 62.34 |  |
| Use sometimes or occasionally | 36 | 24.66 | 548 | 29.57 |  |
| Never use | 7 | 4.79 | 150 | 8.09 |  |
| Number of female partners |  |  |  |  | 0.0028 |
| 0 | 108 | 73.97 | 1571 | 84.78 |  |
| 1 | 33 | 22.60 | 246 | 13.28 |  |
| ≥2 | 5 | 3.43 | 36 | 1.94 |  |
| Internet dating |  |  |  |  | <0.0001 |
| No | 33 | 22.60 | 722 | 38.96 |  |
| Yes | 113 | 77.40 | 1131 | 61.04 |  |
| Alcohol use |  |  |  |  | 0.6878 |
| Every day | 8 | 5.48 | 82 | 4.43 |  |
| At least 3 times per week | 16 | 10.96 | 148 | 7.99 |  |
| At least 1 time per week | 21 | 14.38 | 262 | 14.14 |  |
| Less than 1 time per week | 54 | 36.99 | 702 | 37.88 |  |
| Never drink | 47 | 32.19 | 659 | 35.56 |  |
| Commercial sexual services |  |  |  |  | 0.0859 |
| No | 135 | 92.47 | 1771 | 95.57 |  |
| Yes | 11 | 7.53 | 82 | 4.43 |  |
| Recreational drug use |  |  |  |  | 0.9681^ |
| No | 143 | 97.95 | 1811 | 97.73 |  |
| Yes | 3 | 2.05 | 42 | 2.27 |  |
| HIV status |  |  |  |  | <0.0001 |
| Negative | 132 | 90.41 | 1795 | 96.87 |  |
| Positive | 14 | 9.59 | 58 | 3.13 |  |

HIV: Human immunodeficiency virus; RMB: the legal tender of China, the unit is Yuan; AIDS: Acquired immune deficiency syndrome; STD: Sexually transmitted diseases.

^：Fisher exact probability test.

The LASSO variable screening process for the HIV and STD prediction models were shown in **Figure S2**. A total of 15 variables were screened for the HIV prediction model based on the LASSO variable screening results: degree of education, employment situation, monthly disposable income, HIV knowledge score, HIV testing, HIV counseling, perceived AIDS severity, perceived proportion of AIDS infections, and number of male partners, high-risk oral sex, condom use, Internet dating, commercial sexual service, recreational drug use, and STD history.

The STD prediction model screened for a total of nine variables: degree of education, employment situation, HIV testing, number of male partners, high-risk oral sex, condom use, number of female partners, Internet dating, commercial sexual service, and HIV status.


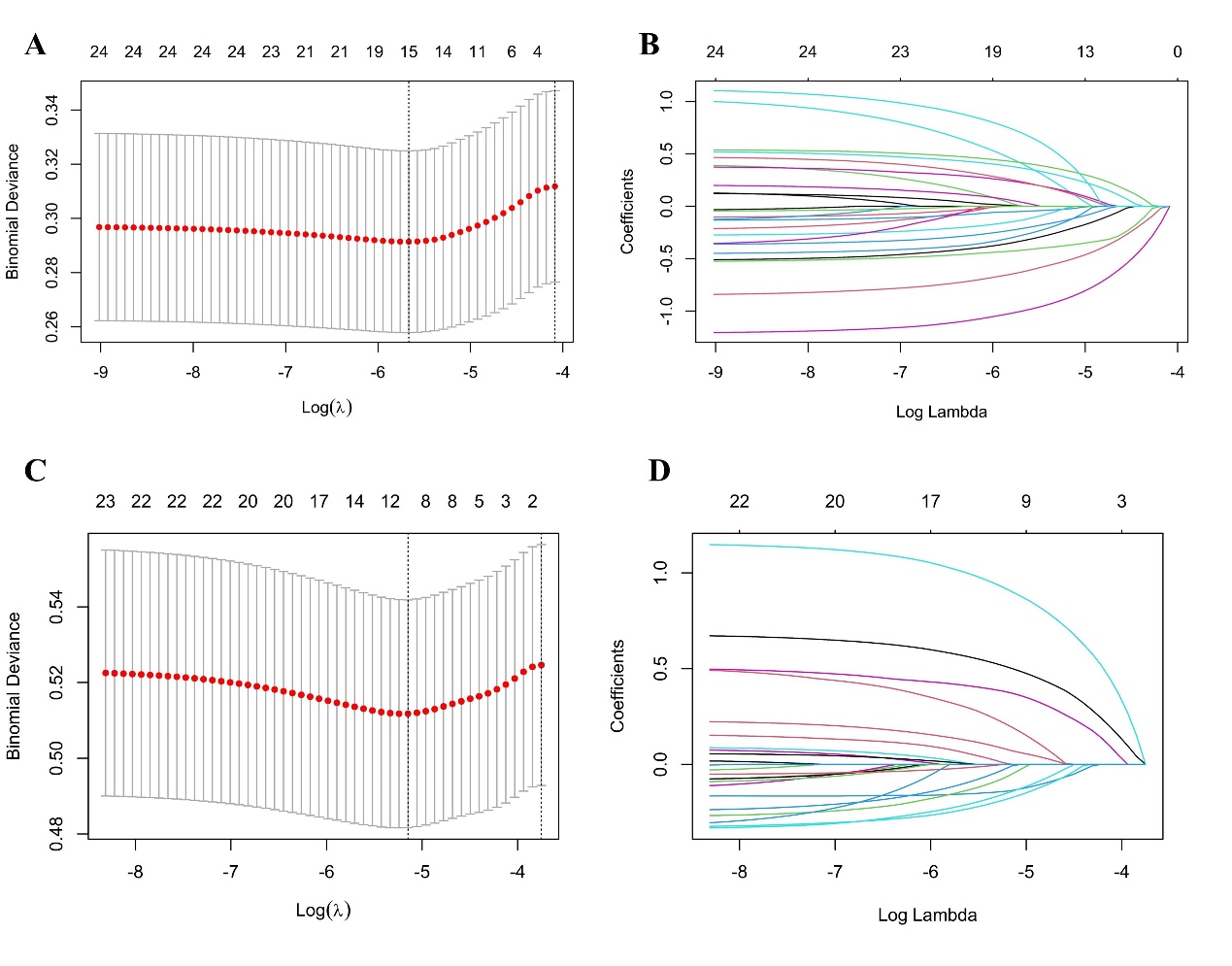


**Figure S2. LASSO-based variable screening process for HIV and STD prediction models**

A: Variable screening for LASSO in HIV prediction model;

B: Regression coefficients for LASSO in HIV prediction model;

C: Variable screening for LASSO in STD prediction model;

D: Regression coefficients for LASSO in STD prediction model.

The Boruta variable screening process for the HIV and STD prediction models were shown in **Figure S3**. Based on the results, variables were selected that were determined to be confirmed and tentative, and a total of 10 variables were included in the HIV prediction model: age, HIV knowledge score, HIV testing, sexual role, number of male partners, high-risk anal sex, condom use, Internet dating, recreational drug use, and STD history.

A total of 15 variables were included in the STD prediction model: age, household registration location, degree of education, employment situation, marital status, monthly disposable income, HIV testing, HIV counseling, perceived proportion of AIDS infections, sexual role, number of male partners, condom use, number of female partners, Internet dating, and commercial sexual service.


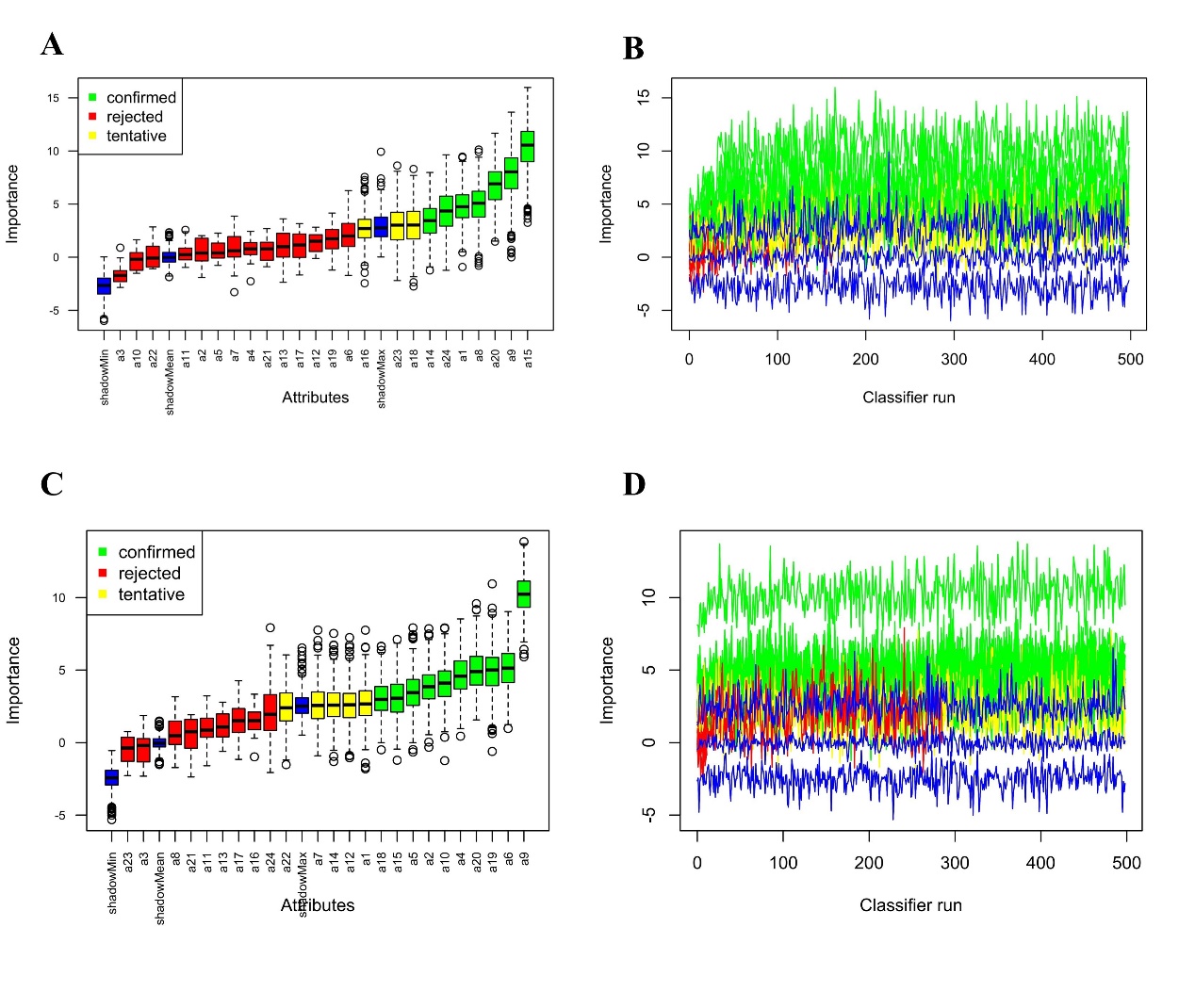


**Figure S3. Boruta-based variable screening process for HIV and STD prediction models**

A: Variable screening of Boruta in HIV prediction model;

B: Stability over 500 iterations in HIV prediction model;

C: Variable screening of Boruta in STD prediction model;

D: Stability over 500 iterations in STD prediction model.

Based on the results of the statistical analysis, a total of seven predictors were finally included in the HIV prediction model: HIV knowledge score, HIV testing, number of male partners, condom use, Internet dating, recreational drug use, and STD history. A total of five predictors were included in the STD prediction model: degree of education, number of male partners, number of female partners, Internet dating, and commercial sexual service.

**3. Comparison of training and testing sets for the prediction model**

A comparison of the training and the testing sets in the HIV prediction model was shown in **Table S3**. According to the results, it could be concluded that there was no statistical difference between the training set and testing set of each variable in the model for predicting the risk of HIV infection.

**Table S3. Comparison of training and testing sets for HIV prediction model**

| Variables | Training set  (N=1499) | | Testing set  (N=500) | | p-value |
| --- | --- | --- | --- | --- | --- |
|  | N | % | N | % |  |
| HIV knowledge scores |  |  |  |  | 0.6677 |
| <11 | 826 | 55.10 | 270 | 54.00 |  |
| ≥11 | 673 | 44.90 | 230 | 46.00 |  |
| HIV testing |  |  |  |  | 0.9364 |
| No | 209 | 13.94 | 69 | 13.80 |  |
| Yes | 1290 | 86.06 | 431 | 86.20 |  |
| Number of male partners |  |  |  |  | 0.5994 |
| 0 | 224 | 14.94 | 74 | 14.80 |  |
| 1 | 840 | 56.04 | 292 | 58.40 |  |
| ≥2 | 435 | 29.02 | 134 | 26.80 |  |
| Condom use |  |  |  |  | 0.0840 |
| Use every time | 929 | 61.97 | 329 | 65.80 |  |
| Use sometimes or occasionally | 457 | 30.49 | 127 | 25.40 |  |
| Never use | 113 | 7.54 | 44 | 8.80 |  |
| Internet dating |  |  |  |  | 0.3294 |
| No | 557 | 37.16 | 198 | 39.60 |  |
| Yes | 942 | 62.84 | 302 | 60.40 |  |
| Recreational drug use |  |  |  |  | 0.1924 |
| No | 1469 | 98.00 | 485 | 97.00 |  |
| Yes | 30 | 2.00 | 15 | 3.00 |  |
| STD history |  |  |  |  | 0.0589 |
| No | 1380 | 92.06 | 473 | 94.60 |  |
| Yes | 119 | 7.94 | 27 | 5.40 |  |
| HIV status |  |  |  |  | 0.7797 |
| Negative | 1444 | 96.33 | 483 | 96.60 |  |
| Positive | 55 | 3.67 | 17 | 3.40 |  |

HIV: Human immunodeficiency virus; STD: Sexually transmitted diseases.

A comparison of the training and the testing sets in the STD prediction model was shown in **Table S4**. According to the results, it could be concluded that there was no statistical difference between the training set and testing set of each variable in the model for predicting the risk of STD infection.

**Table S4. Comparison of training and testing sets for STD prediction model**

| Variables | Training set  (N=1499) | | Testing set  (N=500) | | p-value |
| --- | --- | --- | --- | --- | --- |
|  | N | % | N | % |  |
| Degree of education |  |  |  |  | 0.2522 |
| Primary education and lower | 30 | 2.00 | 12 | 2.40 |  |
| Junior high school | 122 | 8.14 | 48 | 9.60 |  |
| Senior high school | 765 | 51.03 | 230 | 46.00 |  |
| Bachelor’s degree or higher | 582 | 38.83 | 210 | 42.00 |  |
| Number of male partners |  |  |  |  | 0.1972 |
| 0 | 214 | 14.27 | 84 | 16.80 |  |
| 1 | 865 | 57.71 | 267 | 53.40 |  |
| ≥2 | 420 | 28.02 | 149 | 29.80 |  |
| Number of female partners |  |  |  |  | 0.6826 |
| 0 | 1253 | 83.59 | 426 | 85.20 |  |
| 1 | 215 | 14.34 | 64 | 12.80 |  |
| ≥2 | 31 | 2.07 | 10 | 2.00 |  |
| Internet dating |  |  |  |  | 0.9020 |
| No | 565 | 37.69 | 190 | 38.00 |  |
| Yes | 934 | 62.31 | 310 | 62.00 |  |
| Commercial sexual services |  |  |  |  | 0.9488 |
| No | 1429 | 95.33 | 477 | 95.40 |  |
| Yes | 70 | 4.67 | 23 | 4.60 |  |
| STD history |  |  |  |  | 0.6172 |
| No | 1387 | 92.53 | 466 | 93.20 |  |
| Yes | 112 | 7.47 | 34 | 6.80 |  |

STD: Sexually transmitted diseases.

**4. Confusion matrix of each prediction model**

The confusion matrix for the HIV prediction model was shown in **Figure S4**, and the confusion matrix for the STD prediction model was shown in **Figure S5**.


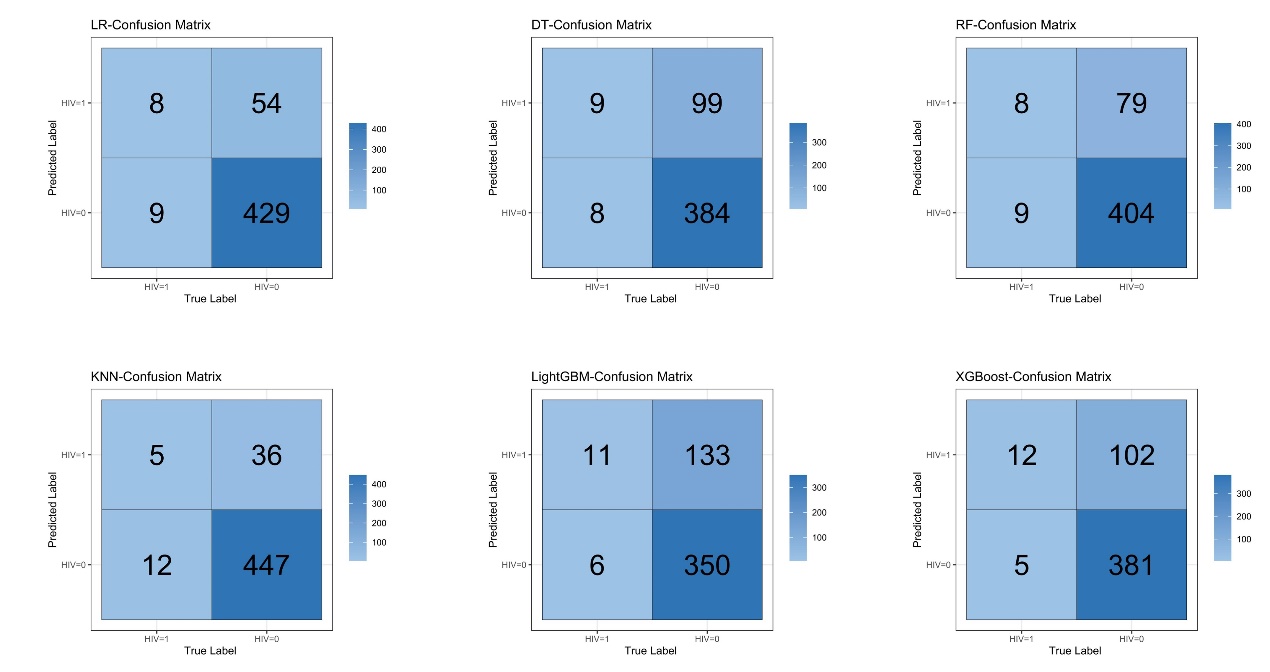


**Figure S4. The confusion matrix for the HIV prediction model**

LR: Logistic regression; DT: Decision tree; RF: Random forest; KNN: K-nearest neighbor; LightGBM: Light gradient boosting machine; XGBoost: eXtreme gradient boosting.


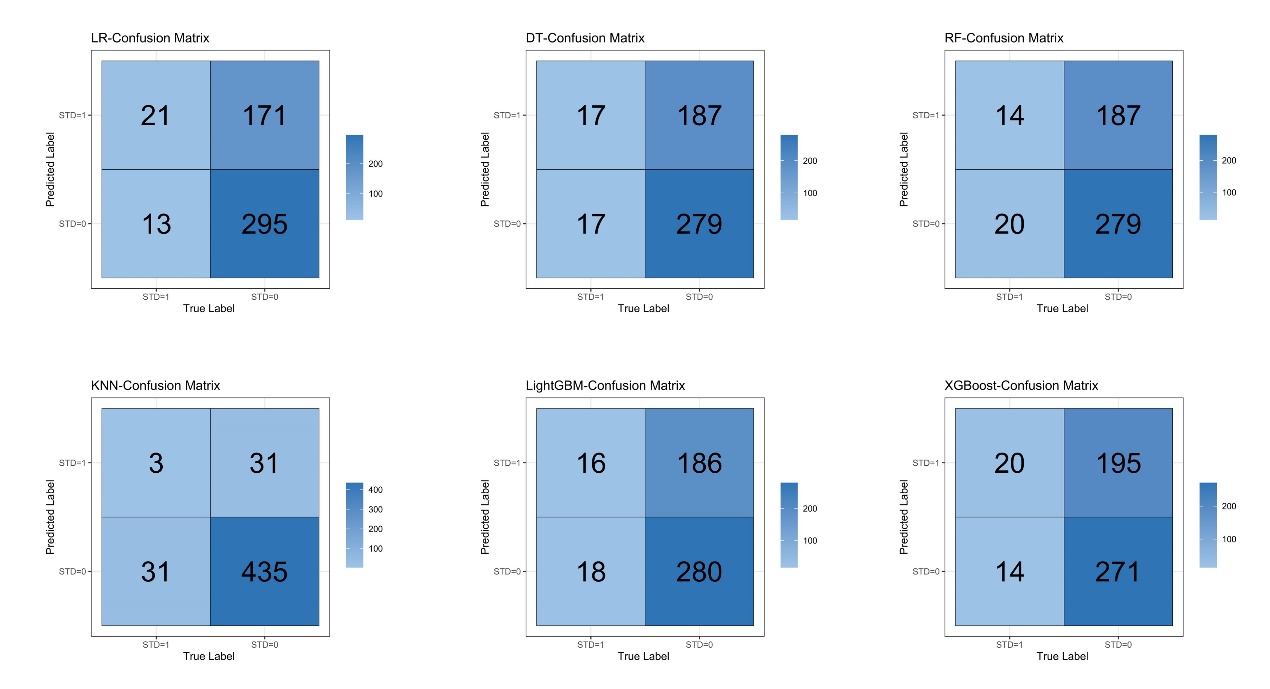


**Figure S5. The confusion matrix for the STD prediction model**

LR: Logistic regression; DT: Decision tree; RF: Random forest; KNN: K-nearest neighbor; LightGBM: Light gradient boosting machine; XGBoost: eXtreme gradient boosting.

**5. Comparison of external and internal validation sets**

Using data from the Sichuan MSM as the external validation set, the differences in demographic characteristics between the external and internal validation sets were shown in **Table S5**. According to the results, age, household registration location, ethnicity, degree of education, and employment situation were statistically different in the internal and external validation sets.

**Table S5. Differences in demographic characteristics between external and internal validation sets**

| Variables | Internal validation sets  (N=1999) | | External validation sets  (N=161) | | p-value |
| --- | --- | --- | --- | --- | --- |
|  | N | % | N | % |  |
| Age |  |  |  |  | 0.0003 |
| 18-25 | 147 | 7.35 | 0 | 0.00 |  |
| 25-35 | 732 | 36.62 | 48 | 29.81 |  |
| 35-45 | 689 | 34.47 | 70 | 43.48 |  |
| ≥45 | 431 | 21.56 | 43 | 26.71 |  |
| Household registration location |  |  |  |  | 0.0193 |
| Urban | 1430 | 71.54 | 129 | 80.12 |  |
| Rural | 569 | 28.46 | 32 | 19.88 |  |
| Ethnicity |  |  |  |  | 0.0359 |
| Han | 1831 | 91.60 | 155 | 96.27 |  |
| Ethnic minorities | 168 | 8.40 | 6 | 3.73 |  |
| Degree of education |  |  |  |  | 0.0209 |
| Primary education and lower | 42 | 2.10 | 8 | 4.97 |  |
| Junior high school | 170 | 8.50 | 6 | 3.73 |  |
| Senior high school | 995 | 49.77 | 79 | 49.07 |  |
| Bachelor’s degree or higher | 792 | 39.63 | 68 | 42.23 |  |
| Employment situation |  |  |  |  | 0.0114 |
| Unemployed | 177 | 8.85 | 24 | 14.91 |  |
| Employed | 1591 | 79.59 | 113 | 70.19 |  |
| Student | 231 | 11.56 | 24 | 14.91 |  |
| Marital status |  |  |  |  | 0.8824 |
| Unmarried | 1685 | 84.29 | 135 | 83.85 |  |
| Married | 314 | 15.71 | 26 | 16.15 |  |
| Monthly disposable income |  |  |  |  | 0.1336 |
| 1000-3000 RMB | 811 | 40.57 | 76 | 47.20 |  |
| 3000-10000 RMB | 1088 | 54.43 | 81 | 50.32 |  |
| ≥10000 RMB | 100 | 5.00 | 4 | 2.48 |  |

RMB: the legal tender of China, the unit is Yuan.

The differences between the external and internal validation sets for each variable in the HIV prediction model were shown in **Table S6**. According to the results, number of male partners, condom use, and HIV infection status were statistically different in the internal and external validation sets.

**Table S6. Comparison of variables in the internal and external validation sets in the HIV prediction model**

| Variables | Internal validation sets  (N=1999) | | External validation sets  (N=161) | | p-value |
| --- | --- | --- | --- | --- | --- |
|  | N | % | N | % |  |
| HIV knowledge scores |  |  |  |  | 0.9117 |
| <11 | 1096 | 54.83 | 89 | 55.28 |  |
| ≥11 | 903 | 45.17 | 72 | 44.72 |  |
| HIV testing |  |  |  |  | 0.1577 |
| No | 278 | 13.91 | 16 | 9.94 |  |
| Yes | 1721 | 86.09 | 145 | 90.06 |  |
| Number of male partners |  |  |  |  | 0.0047 |
| 0 | 298 | 14.91 | 9 | 5.59 |  |
| 1 | 1132 | 56.63 | 103 | 63.98 |  |
| ≥2 | 569 | 28.46 | 49 | 30.43 |  |
| Condom use |  |  |  |  | 0.0126 |
| Use every time | 1258 | 62.93 | 83 | 51.55 |  |
| Use sometimes or occasionally | 584 | 29.22 | 64 | 39.75 |  |
| Never use | 157 | 7.85 | 14 | 8.70 |  |
| Internet dating |  |  |  |  | 0.6604 |
| No | 755 | 37.77 | 58 | 36.02 |  |
| Yes | 1244 | 62.23 | 103 | 63.98 |  |
| Recreational drug use |  |  |  |  | 0.4879 |
| No | 1954 | 97.75 | 156 | 96.89 |  |
| Yes | 45 | 2.25 | 5 | 3.11 |  |
| STD history |  |  |  |  | 0.8246 |
| No | 1853 | 92.70 | 150 | 6.83 |  |
| Yes | 146 | 7.30 | 11 | 93.17 |  |
| HIV status |  |  |  |  | 0.0402 |
| Negative | 1927 | 96.40 | 150 | 93.17 |  |
| Positive | 72 | 3.60 | 11 | 6.83 |  |

HIV: Human immunodeficiency virus; STD: Sexually transmitted diseases.

Differences between the external and internal validation sets for each variable in the STD prediction model were shown in **Table S7**. According to the results, degree of education, number of male partners, and commercial sexual service were statistically different in the internal and external validation sets.

**Table S7. Comparison of variables in the internal and external validation sets in the STD prediction model**

| Variables | Internal validation sets  (N=1999) | | External validation sets  (N=161) | | p-value |
| --- | --- | --- | --- | --- | --- |
|  | N | % | N | % |  |
| Degree of education |  |  |  |  | 0.0209 |
| Primary education and lower | 42 | 2.10 | 8 | 4.97 |  |
| Junior high school | 170 | 8.50 | 6 | 3.73 |  |
| Senior high school | 995 | 49.77 | 79 | 49.07 |  |
| Bachelor’s degree or higher | 792 | 39.63 | 68 | 42.23 |  |
| Number of male partners |  |  |  |  | <0.0001 |
| 0 | 298 | 14.91 | 1 | 0.62 |  |
| 1 | 1132 | 56.63 | 104 | 64.60 |  |
| ≥2 | 569 | 28.46 | 56 | 34.78 |  |
| Number of female partners |  |  |  |  | 0.1016 |
| 0 | 1679 | 83.99 | 145 | 90.06 |  |
| 1 | 279 | 13.96 | 15 | 9.32 |  |
| ≥2 | 41 | 2.05 | 1 | 0.62 |  |
| Internet dating |  |  |  |  | 0.6604 |
| No | 755 | 37.77 | 58 | 36.02 |  |
| Yes | 1244 | 62.23 | 103 | 63.98 |  |
| Commercial sexual services |  |  |  |  | 0.0090 |
| No | 1906 | 95.35 | 146 | 90.68 |  |
| Yes | 93 | 4.65 | 15 | 9.32 |  |
| STD history |  |  |  |  | 0.8246 |
| No | 1853 | 92.70 | 150 | 93.17 |  |
| Yes | 146 | 7.30 | 11 | 6.83 |  |

STD: Sexually transmitted diseases.

**6. Sensitivity analysis**

The optimal model (XGBoost) was applied to the MMSM and Non-MMSM, MSMO and MSMW datasets, respectively, and the results of the sensitivity analyses were shown in **Figure S6**. Based on the results of the sensitivity analysis, the XGBoost model was able to predict the risk of HIV and STD infection in the Non-MSMM and MSMM subgroups (AUC: 0.634-0.839). The XGBoost model was able to predict the risk of HIV and STD infection in the MSMO and MSMW subgroups (AUC: 0.641-0.797).


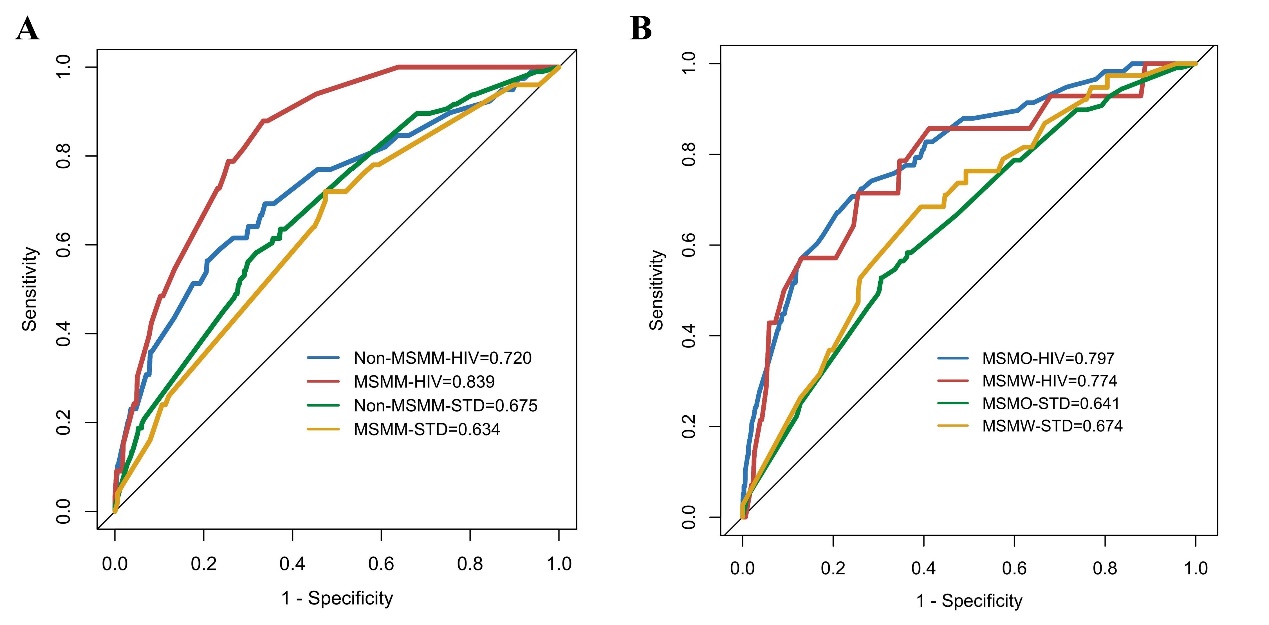


**Figure S6. Results of sensitivity analysis**

A: Performance of optimal prediction model in Non-MSMM and MSMM Subgroup;

B: Performance of optimal prediction model in MSMO and MSMW Subgroup.

HIV: Human immunodeficiency virus; STD: Sexually transmitted diseases; MSMM: men who have sex with multiple men; MSMW: men who have sex with men and women; MSMO: men who have sex with men only.

**7. Evaluating the bias of the data to the population**

It is very important to evaluate the bias of the data to the population. We plan to assess these four aspects. Firstly, regarding sample selection bias, the basic demographic characteristics of the 1999 MSM were described in **Table 1**. The highest percentage of MSM in the recruited population was 25-35 years old at 36.62%, and recruitment was generally among younger MSM. The urban population was relatively high (71.54%), mostly Han ethnicity (91.60%), with a high level of education. The majority of MSM were employed (79.59%), most self-reported as unmarried (84.29%), and fewer were in higher income groups (5.00%). The basic demographic characteristics of the recruiting population showed that our study population did have some bias. However, it has been mentioned in previous studies that the recruitment and sampling of participants in social surveys and studies involving gender and sexual minorities is often extremely challenging. Most research participants tend to conceal their true sexual orientation or gender identity to avoid exposure, and may feel uncomfortable or wary towards the researchers. For such a bias is unavoidable in our study, for which we have explained in our Discussion section. The second concerns self-selection bias; our study relied on voluntary participation by MSM, and the characteristics of participants may differ from non-participants, which may affect the generalizability of the results. This was also explained in our Discussion section. Thirdly, regarding measurement bias; the validity of risk prediction models depended on the accuracy of self-reported information, which was influenced by MSM recall bias and social desirability bias. In future research on MSM, some accurate and reliable information gathering tools are necessary, for example, computer-assisted self-interview (CASI). This is a point of content we have already mentioned in our Discussion section. Finally, regarding the processing of missing data, after a preliminary analysis, all variables had a missing rate of less than 10%, so we used the multiple imputation method to fill in the missing values. In summary, we have assessed the issue of the representativeness of the study population from 4 aspects, and it is true that we cannot avoid some bias for the study population, especially for the specific population of MSM. However, we have proved the reliability of the study results through preliminary analysis. At the same time, we have also explained in our Limitations.

Our study recruited eligible MSM in Western China (Chongqing, Xinjiang, Guangxi, and Sichuan). The number of recruits in each region and the geographical characteristics of the recruitment area were shown in **Figure S7**.


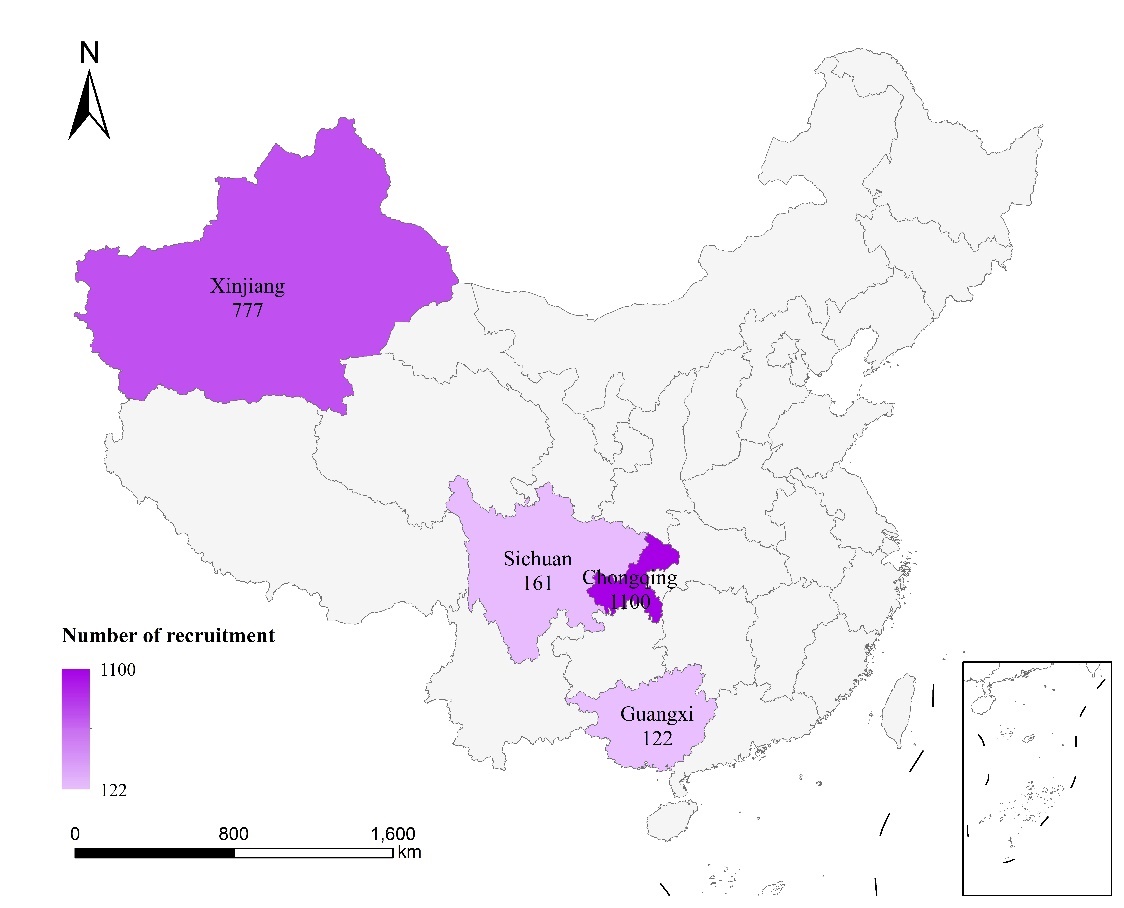


**Figure S7. Geographic distribution of the MSM recruited in this study**

According to Supplementary Figure 7, we recruited 1100, 777, 122, and 161 MSM from Chongqing, Xinjiang, Guangxi, and Sichuan, respectively, with 161 MSM from Sichuan serving as an external validation set. We analyzed the basic demographic differences of MSM from different regions of Western China, as shown in **Table S8**. According to the results of the Chi-square test, except marital status, the other demographic characteristics of MSM in the four regions have statistical differences. The age of MSM in Xinjiang was younger and that in Guangxi was older. Sichuan recruited more urban population, Guangxi recruited more ethnic minorities, and Xinjiang recruited MSM with higher educational level. Most of the participants recruited in Sichuan were employed, while Guangxi recruited more low-income groups.

**Table S8. The basic demographic differences of MSM from different regions of Western China**

| Variables | Chongqing  (N=1100) | Guangxi  (N=122) | Sichuan  (N=161) | Xinjiang  (N=777) | p-value |
| --- | --- | --- | --- | --- | --- |
|  | N(%) | N(%) | N(%) | N(%) |  |
| Age |  |  |  |  | 0.0025 |
| 18-25 | 79(7.18) | 4(3.28) | 0(0.00) | 64(8.25) |  |
| 25-35 | 389(35.36) | 43(35.25) | 51(31.68) | 297(38.22) |  |
| 35-45 | 386(35.10) | 43(35.25) | 73(45.34) | 257(33.08) |  |
| ≥45 | 246(22.36) | 32(26.22) | 37(22.98) | 159(20.45) |  |
| Household registration location |  |  |  |  | <0.0001 |
| Urban | 744(67.64) | 80(65.57) | 140(86.96) | 595(76.58) |  |
| Rural | 356(32.36) | 42(34.43) | 21(13.04) | 182(23.42) |  |
| Ethnicity |  |  |  |  | <0.0001 |
| Han | 1064(96.73) | 92(75.41) | 134(83.23) | 696(89.58) |  |
| Ethnic minorities | 36(3.27) | 30(24.59) | 27(16.77) | 81(10.42) |  |
| Degree of education |  |  |  |  | <0.0001 |
| Primary education and lower | 31(2.82) | 7(5.74) | 4(2.48) | 8(1.03) |  |
| Junior high school | 112(10.18) | 13(10.66) | 11(6.83) | 40(5.15) |  |
| Senior high school | 556(50.55) | 89(72.95) | 71(44.11) | 358(46.07) |  |
| Bachelor’s degree or higher | 401(36.45) | 13(10.65) | 75(46.58) | 371(47.75) |  |
| Employment situation |  |  |  |  | <0.0001 |
| Unemployed | 112(10.18) | 23(18.85) | 9(5.59) | 57(7.34) |  |
| Employed | 844(76.73) | 81(66.40) | 138(85.71) | 641(82.50) |  |
| Student | 144(13.09) | 18(14.75) | 14(8.70) | 79(10.17) |  |
| Marital status |  |  |  |  | 0.6956 |
| Unmarried | 917(83.36) | 103(84.43) | 138(85.71) | 662(85.20) |  |
| Married | 183(16.64) | 19(15.57) | 23(14.29) | 115(14.80) |  |
| Monthly disposable income |  |  |  |  | <0.0001 |
| 1000-3000 RMB | 510(46.36) | 103(84.43) | 53(32.92) | 221(28.45) |  |
| 3000-10000 RMB | 534(48.55) | 19(15.57) | 103(63.98) | 513(66.02) |  |
| ≥10000 RMB | 56(5.09) | 0(0.00) | 5(3.11) | 43(5.53) |  |
| HIV status |  |  |  |  | <0.0001 |
| Negative | 1048(95.27) | 120(98.36) | 144(89.44) | 765(98.46) |  |
| Positive | 52(4.73) | 2(1.64) | 17(10.56) | 12(1.54) |  |
| STD history |  |  |  |  | <0.0001 |
| No | 1018(92.55) | 106(86.89) | 138(85.71) | 741(95.37) |  |
| Yes | 82(7.45) | 16(13.11) | 23(14.29) | 36(4.63) |  |

RMB: the legal tender of China, the unit is Yuan; HIV: Human immunodeficiency virus; STD: Sexually transmitted diseases.

These differences may be related to the social background characteristics of the region. For example, for the age difference, the younger MSM in Xinjiang may be related to the intensive recruitment of universities, while the older MSM in Guangxi may be related to the different publicity strategies of our partner institutions (local CDC) or the social acceptance. The high proportion of ethnic minorities in Guangxi may reflect the local ethnic diversity, and the income level of MSM in different regions may be related to the local economic level. Our results revealed significant spatial heterogeneity in demographic characteristics of the MSM population in different regions of Western China, which reflected the uneven distribution of socioeconomic, cultural, and health resources. In view of these differences, it is necessary to take into account the regional and population characteristics to carry out regional management policies, design precise health intervention and publicity programs for MSM population in different areas, and optimize resource allocation. For example, strengthening health education for young populations in Xinjiang and optimizing resource support for economically disadvantaged groups in Guangxi. In the future, the impact of these heterogeneity on the prevention effect should be further quantified to provide scientific evidence for optimizing the precise intervention of MSM population. In addition, for the outcome variables (HIV and STD), our results indicated that MSM in Sichuan had higher risk of both HIV and STD infection than those in the other three regions. It is necessary to develop regional, comprehensive and precise prevention and control strategies, and strengthen the in-depth study on social and behavioral factors to provide scientific evidence for comprehensive health intervention in MSM population.

In summary, our study revealed significant spatial heterogeneity in the demographic characteristics and HIV/STD infection risks among the MSM population in Western China. This heterogeneity reflects regional differences in population structure, socioeconomic conditions, behavioral patterns, and access to healthcare services. Considering the epidemiological context, these findings suggest the need to develop tailored prevention and control strategies based on regional characteristics to effectively reduce the risk of HIV and STD transmission in various areas.

**8. Reporting strictly adheres to the Guidelines**

Our study strictly adheres to the “Guidelines for Developing and Reporting Machine Learning Predictive Models in Biomedical Research”. We have carefully read Wei Luo's article published in the Journal of Medical Internet Research. Based on the table in the article, we created a checklist to report adherence to the guidelines. We have ensured that all key components, including model development, evaluation, and reporting, follow the recommendations outlined in the guidelines. This checklist table was shown in **Table S9**.

**Table S9. The checklist to the “Guidelines for Developing and Reporting Machine Learning Predictive Models in Biomedical Research”**

| Number | Section | Topic | Checklist item | Response |
| --- | --- | --- | --- | --- |
| 1 | Title | Nature of study | Identify the report as introducing a predictive model | ☑ |
| 2 | Abstract | Structured summary | Background  Objectives  Data sources  Performance metrics of the predictive model or models, in both point estimates and confidence intervals  Conclusion including the practical value of the developed predictive model or models | ☑ |
| 3 | Introduction | Rationale | Identify the clinical goal  Review the current practice and prediction accuracy of any existing models | ☑ |
| 4 | Introduction | Objectives | State the nature of study being predictive modeling, defining the target of prediction  Identify how the prediction problem may benefit the clinical goal | ☑ |
| 5 | Methods | Describe the setting | Identify the clinical setting for the target predictive model.  Identify the modeling context in terms of facility type, size, volume, and duration of available data. | ☑ |
| 6 | Methods | Define the prediction problem | Define a measurement for the prediction goal (per patient or per hospitalization or per type of outcome).  Determine that the study is retrospective or prospective.  Identify the problem to be prognostic or diagnostic.  Determine the form of the prediction model:  (1) classification if the target variable is categorical,  (2) regression if the target variable is continuous,  (3) survival prediction if the target variable is the time to an event.  Translate survival prediction into a regression problem, with the target measured over a temporal window following the time of prediction.  Explain practical costs of prediction errors (eg, implications of underdiagnosis or overdiagnosis).  Defining quality metrics for prediction models.  Define the success criteria for prediction (eg, based on metrics in internal validation or external validation in the context of the clinical problem). | ☑ |
| 7 | Methods | Prepare data for model building | Identify relevant data sources and quote the ethics approval number for data access.  State the inclusion and exclusion criteria for data.  Describe the time span of data and the sample or cohort size.  Define the observational units on which the response variable and predictor variables are defined.  Define the predictor variables. Extra caution is needed to prevent information leakage from the response variable to predictor variables.  Describe the data preprocessing performed, including data cleaning and transformation.  Remove outliers with impossible or extreme responses; state any criteria used for outlier removal.  State how missing values were handled.  Describe the basic statistics of the dataset, particularly of the response variable. These include the ratio of positive to negative classes for a classification problem and the distribution of the response variable for regression problem.  Define the model validation strategies. Internal validation is the minimum requirement; external validation should also be performed whenever possible.  Specify the internal validation strategy. Common methods include random split, time-based split, and patient-based split.  Define the validation metrics. For regression problems, the normalized root-mean-square error should be used. For classification problems, the metrics should include sensitivity, specificity, positive predictive value, negative predictive value, area under the ROC curve, and calibration plot.  For retrospective studies, split the data into a derivation set and a validation set. For prospective studies, define the starting time for validation data collection. | ☑ |
| 8 | Methods | Build the predictive model | Identify independent variables that predominantly take a single value (eg, being zero 99% of the time).  Identify and remove redundant independent variables.  Identify the independent variables that may suffer from the perfect separation problem.  Report the number of independent variables, the number of positive examples, and the number of negative examples.  Assess whether sufficient data are available for a good fit of the model. In particular, for classification, there should be a sufficient number of observations in both positive and negative classes.  Determine a set of candidate modeling techniques (eg, logistic regression, random forest, or deep learning). If only one type of model was used, justify the decision for using that model.  Define the performance metrics to select the best model.  Specify the model selection strategy. Common methods include K-fold validation or bootstrap to estimate the lost function on a grid of candidate parameter values. For K-fold validation, proper stratification by the response variable is needed.  For model selection, include discussion on (1) balance between model accuracy and model simplicity or interpretability, and (2) the familiarity with the modeling techniques of the end user. | ☑ |
| 9 | Results | Report the final model and performance | Report the predictive performance of the final model in terms of the validation metrics specified in the methods section.  If possible, report the parameter estimates in the model and their confidence intervals. When the direct calculation of confidence intervals is not possible, report nonparametric estimates from bootstrap samples.  Comparison with other models in the literature should be based on confidence intervals.  Interpretation of the final model. If possible, report what variables were shown to be predictive of the response variable. State which subpopulation has the best prediction and which subpopulation is most difficult to predict. | ☑ |
| 10 | Discussion | Clinical implications | Report the clinical implications derived from the obtained predictive performance. For example, report the dollar amount that could be saved with better prediction. How many patients could benefit from a care model leveraging the model prediction? And to what extent? | ☑ |
| 11 | Discussion | Limitations of the model | Discuss the following potential limitations:  • Assumed input and output data format  • Potential pitfalls in interpreting the model  • Potential bias of the data used in modeling  • Generalizability of the data | ☑ |
| 12 | Discussion | Unexpected results during the experiments | Report unexpected signs of coefficients, indicating collinearity or complex interaction between predictor variables | ☑ |
